# Hospitalizations for congenital infections in Brazil’s unified health system: nationwide trends and regional disparities, 2008–2024

**DOI:** 10.1017/ash.2026.10300

**Published:** 2026-02-03

**Authors:** Gustavo Yano Callado, Marina Martins Siqueira, Lucas Hernandes Corrêa, Felipe Mendes Delpino, Alexandre R. Marra, Eduardo Félix Martins Santana

**Affiliations:** 1 Faculdade Israelita de Ciências da Saúde Albert Einstein, Hospital Israelita Albert Einstein, São Paulo, Brazil; 2 Centro de Estudos e Promoção de Políticas de Saúde (CEPPS), Sociedade Beneficente Israelita Brasileira Albert Einstein, São Paulo, Brazil; 3 Department of Internal Medicine, University of Iowa Health Care, Iowa City, IA, USA; 4 Department of Obstetrics, Federal University of São Paulo (UNIFESP), São Paulo, Brazil

## Abstract

**Background:**

Congenital infections cause stillbirth, prematurity, birth defects, and neonatal death, representing a major preventable cause of infant morbidity and mortality. In Brazil, data on their hospital burden remain limited.

**Methods:**

This retrospective, population-based time series study analyzed hospitalizations of infants (<12 mo) primarily associated with congenital syphilis, toxoplasmosis, rubella, cytomegalovirus, or herpes in Brazil’s Unified Health System (SUS) from 2008 to 2024. Data were extracted from the SUS Hospital Information System (SIH). Hospitalizations were evaluated by annual volume, population-adjusted rates, mean and total costs, intensive care unit (ICU) use, length of stay (LOS), and in-hospital mortality, stratified by region. Temporal trends were examined using Spearman’s correlation and group differences using one-way ANOVA.

**Results:**

A total of 194,531 hospitalizations were recorded, representing a 394% increase from 4,449 in 2008 to 20,971 in 2024. Congenital syphilis accounted for 88% of admissions and increased across all regions, while toxoplasmosis and cytomegalovirus rose moderately and rubella declined following immunization. National hospital expenditures reached US$49.1 million, rising 170% over the period. Mean LOS decreased modestly (−.8 d), and ICU use remained low except for herpes (up to 32%). In-hospital mortality declined from .73% to .13%, but 29.5% of patients were hospitalized outside their municipality of residence, indicating persistent regional disparities.

**Conclusions:**

Hospitalizations due to congenital infections—predominantly syphilis—have increased substantially in Brazil, reflecting gaps in prenatal screening, partner management, and maternal–child health coordination. Despite declining mortality, regional inequalities in hospitalization rates, access, and costs persist.

## Graphical abstract

**Figure:**
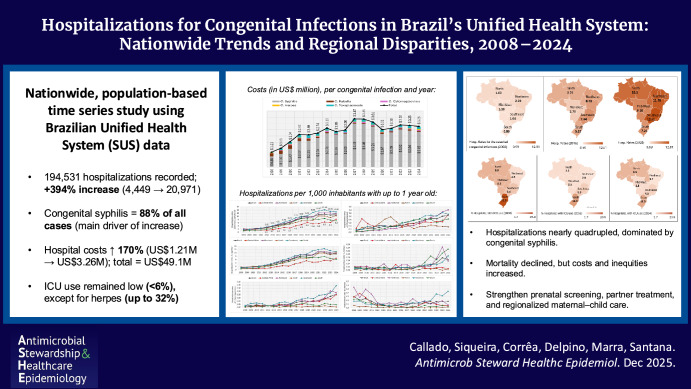

## Introduction

Congenital infections cause significant social and financial burdens for families and healthcare systems because they are linked to stillbirth, prematurity, congenital malformations, sensory impairment, and neonatal death.^1–4^ Congenital syphilis has significantly increased in Brazil in recent decades, which is indicative of ongoing shortcomings in prenatal screening, prompt treatment, and high-quality prenatal care, particularly in socioeconomically disadvantaged groups.^1,4^ Even though they are less common, other congenital infections like toxoplasmosis, rubella, cytomegalovirus, and herpes also cause hospital stays, neurodevelopmental consequences, and avoidable infant deaths, making them crucial targets for neonatal care and surveillance.^1,3^

Routine prenatal serological screening, antimicrobial therapy, rubella vaccination, and neonatal management protocols are among the prevention and treatment strategies implemented by the Brazilian Unified Health System (*Sistema Único de Saúde*, SUS).^5,6^ Congenital syphilis, however, is still on the rise, with significant regional disparities in access to testing and prompt treatment for mothers and partners.^5,7^ Although rubella, cytomegalovirus, and herpes persist at smaller scales but can cause serious neonatal complications requiring specialized care, congenital toxoplasmosis is still endemic in several areas with limited prenatal screening.^3,4^

Despite their clinical relevance, knowledge about the overall hospital impact of these infections in Brazil remains limited. Most studies focus on congenital syphilis, restricted to specific regions or short time frames.^8–10^ Nationwide assessments of hospitalizations, costs, intensive care use, length of stay, mortality, and regional disparities across these five infections are lacking. This gap hampers accurate burden estimation and the development of integrated prevention and care strategies.^3,8,10^

Therefore, this study conducted a population-based analysis of hospitalizations associated with congenital syphilis, toxoplasmosis, rubella, cytomegalovirus, and herpes in Brazil (2008–2024), describing trends, costs, hospitalization rates, intensive care utilization, length of stay, in-hospital mortality, and regional disparities within SUS. The findings provide evidence to guide public health policies and resource allocation for congenital infection prevention and care nationwide.

## Methods

This retrospective, population-based time series study analyzed hospitalizations associated with congenital infections within SUS from 2008 to 2024. The data set was de-identified and publicly available from the SUS Hospital Information System (SIH). Data collection occurred in July 2025 using the SUS desktop tabulator (TABWIN) and was organized into Microsoft Excel spreadsheets.

We included hospitalizations of participants younger than 12 months of age primarily associated with five congenital infections, identified through the International Classification of Diseases, 10^th^ Revision (ICD-10)^11^: congenital syphilis (codes A50, A50.0–A50.9), rubella (code P35.0), cytomegalovirus (code P35.1), herpes (code P35.2), and toxoplasmosis (code P37.1). Case notification within SIH integrates laboratory evidence (eg, serology or pathogen detection), compatible clinical findings in the newborn, and maternal epidemiological history.

Hospitalizations were characterized by their annual volume, population-adjusted rates, total and mean costs, ICU use, intra-hospital length of stay (LOS), and in-hospital mortality. To calculate hospitalization rates, the population under 1 year of age covered by health plans, based on data from the National Supplementary Health Agency (ANS),^12^ was subtracted from the population under 1 year of age projected by the Brazilian Institute of Geography and Statistics (IBGE).^13^ Results were stratified by congenital infection type and by Brazilian region where the hospitalization occurred. The hospitalization costs, provided in Brazilian Real (R$) were converted to US Dollars (US$) using annual commercial exchange rates from the Central Bank of Brazil, as provided by the Institute for Applied Economic Research (IPEA).^14^ These cost values correspond to reimbursements established by SUS based on standardized procedure codes and fixed compensation rates, which may not reflect the actual economic cost of hospitalization.

This study did not require institutional review board approval or patient informed consent because it analyzed secondary, publicly available, and de-identified data from SIH/SUS, in accordance with national and international ethical standards.

### Statistical analysis

Hospitalization rates, average costs, mean lengths of stay, and in-hospital mortality were compared among Brazilian regions and congenital infection types using one-way ANOVA (Supplementary Material–Statistical Analyses). Spearman’s rank correlation was used to evaluate temporal variations from 2008 to 2024 in order to look for relationships between time and the variables under study. *T*-tests were then used to determine the significance of these correlations. Stata 14.1 (College Station, TX) was used for all analyses, with *α* = 0.05.

For the analysis of patient flows, municipal identifiers (IBGE unique codes) were converted into geographic data in Excel, and the latitude and longitude of each municipality pair (patient’s residence and hospital location) were obtained. Straight-line distances between municipalities were then calculated using the Haversine formula, which accounts for Earth’s curvature based on latitude and longitude coordinates.

## Results

### Number of hospitalizations

Brazil’s hospitalization rate for congenital infections rose gradually between 2008 and 2021, especially between 2012 and 2018, before slightly declining after 2021 (Table 1). Hospitalizations increased from 4,449 in 2008 to 20,971 in 2024, a 394% increase. From 3,371 cases in 2009 (75.8%) to over 20,000 cases per year in 2021–2022 (averaging 88% of hospitalizations), congenital syphilis predominated. After 2010, rubella-related admissions significantly decreased as a result of widespread vaccination. Herpes and cytomegalovirus were rare (<2% combined), and toxoplasmosis stayed steady (7–12%).

**Table 1.** Number of hospitalizations, per year and congenital infection

	Syphilis	Toxoplasmosis	Rubella	Cytomegalovirus	Herpes	Total
**2008**	3,136 (73.9)	280 (6.6)	735 (17.3)	55 (1.3)	37 (.9)	4,243
**2009**	3,371 (75.8)	469 (10.5)	507 (11.4)	69 (1.6)	33 (.7)	4,449
**2010**	3,680 (74.1)	595 (12.0)	569 (11.5)	93 (1.9)	31 (.6)	4,968
**2011**	4,556 (81.0)	561 (10.0)	411 (7.3)	74 (1.3)	27 (.5)	5,629
**2012**	5,539 (84.2)	723 (11.0)	224 (3.4)	58 (.9)	31 (.5)	6,575
**2013**	7,125 (86.2)	859 (10.4)	169 (2.0)	65 (.8)	45 (.5)	8,263
**2014**	9,047 (88.3)	919 (9.0)	162 (1.6)	99 (1.0)	14 (.1)	10,241
**2015**	11,518 (90.7)	893 (7.0)	147 (1.2)	108 (.9)	34 (.3)	12,700
**2016**	12,819 (90.3)	1,034 (7.3)	200 (1.4)	98 (.7)	40 (.3)	14,191
**2017**	15,222 (90.7)	1,172 (7.0)	167 (1.0)	174 (1.1)	41 (.2)	16,776
**2018**	18,075 (91.1)	1,253 (6.3)	289 (1.5)	210 (1.1)	24 (.1)	19,851
**2019**	18,140 (90.2)	1,491 (7.4)	216 (1.1)	213 (1.1)	52 (.3)	20,112
**2020**	18,692 (89.3)	1,709 (8.2)	294 (1.4)	196 (.9)	56 (.3)	20,947
**2021**	20,962 (89.8)	1,833 (7.9)	236 (1.0)	229 (1.0)	71 (.3)	23,331
**2022**	20,349 (89.5)	1,691 (7.4)	334 (1.5)	271 (1.2)	91 (.4)	22,736
**2023**	18,300 (86.8)	1,967 (9.3)	342 (1.6)	339 (1.6)	151 (.7)	21,099
**2024**	18,045 (86.0)	2,071 (9.9)	333 (1.6)	395 (1.9)	127 (.6)	20,971
**Total**	20,8576 (88.0)	19,520 (8.2)	5,335 (2.3)	2,746 (1.2)	905 (.4)	237,08

*Source*: Developed by authors, with data extracted from SIH/SUS, considering the selected ICD-10 codes.

Data are presented as absolute numbers with annual percentages in parentheses.

**Figure 1.**
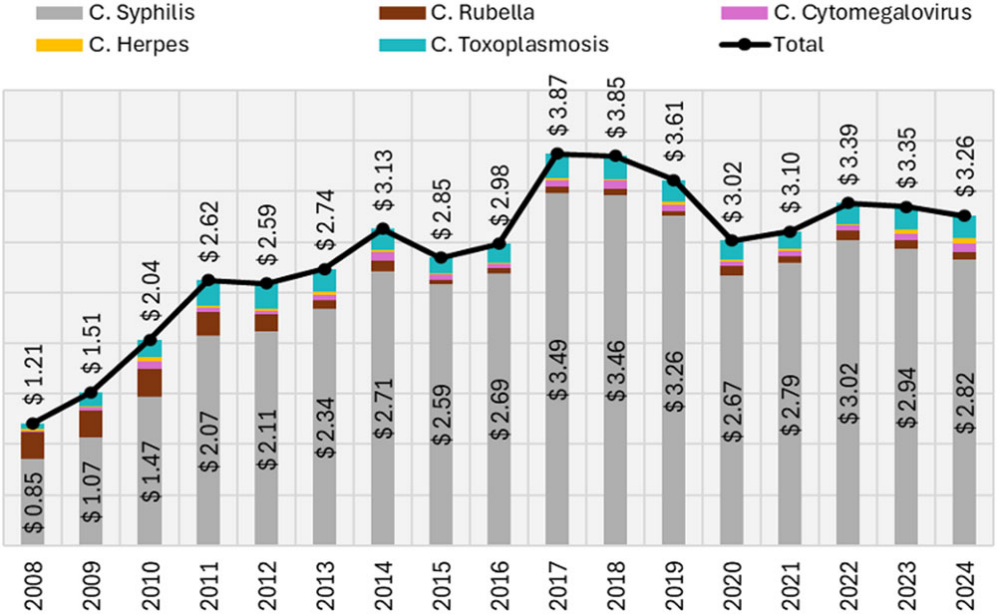
Total SUS’ reimbursement (in US$ million), per congenital infection and year. Source: Developed by authors, with data extracted from SIH/SUS, considering the selected ICD-10 codes. Values in Brazilian Real (R$) were converted to US Dollars (US$) using annual commercial exchange rates from the Central Bank of Brazil (BACEN), as provided by the Institute for Applied Economic Research (IPEA): https://www.ipeadata.gov.br/ExibeSerie.aspx?serid=31924.

In 2024, congenital syphilis caused 18,045 hospitalizations, compared with 127 for herpes. Most infections showed a significant upward trend; rubella was the exception, decreasing from 2008 to 2017, fluctuating from 2018 to 2021, and stabilizing thereafter (S. Figure 1).

### Hospitalization costs

**Figure 2.**
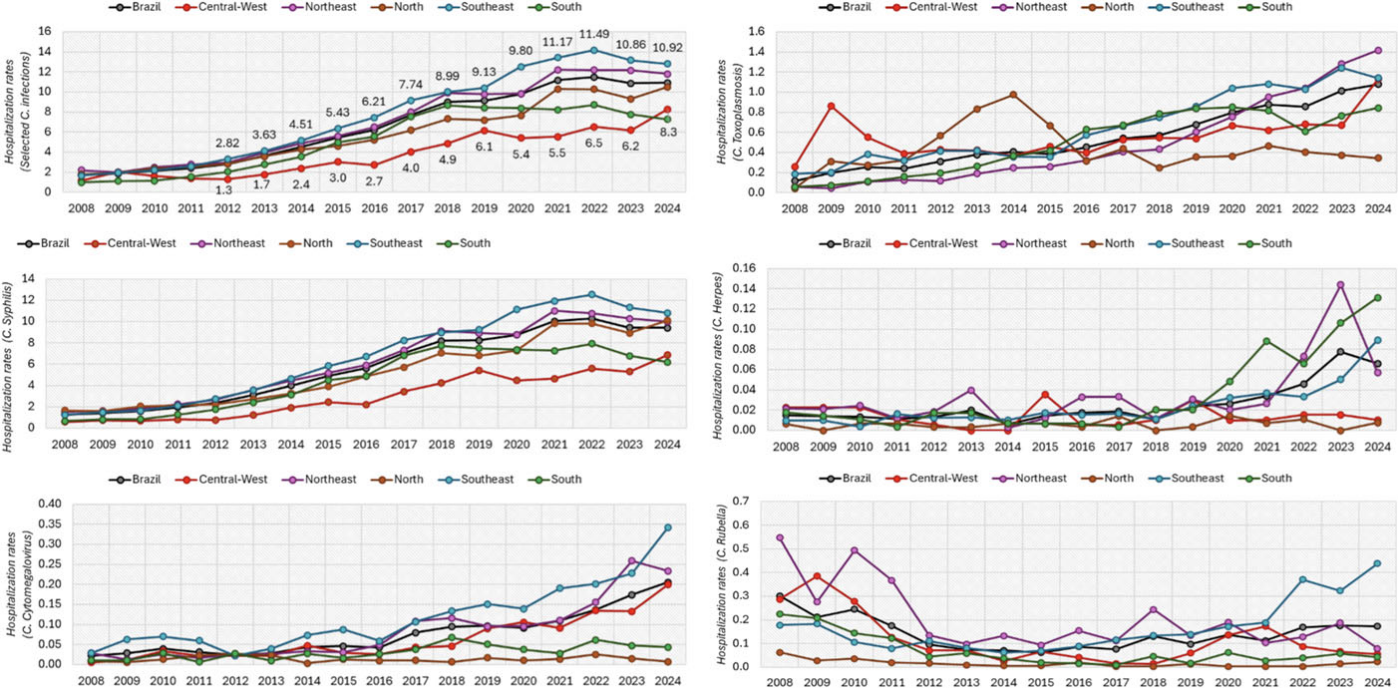
Hospitalizations per 1,000 inhabitants with up to 1 year old and no coverage by private health insurance. Source: developed by authors, with data extracted from SIH/SUS, considering the selected ICD-10 codes.

From 2008 to 2024, SUS spent $49.11 million on hospitalizations for these infections. Annual costs rose from $1.21 million in 2008 to $3.26 million in 2024 (170% increase), driven mainly by congenital syphilis and toxoplasmosis (Figure 1). Average reimbursement per hospitalization was highest for herpes ($444.40), followed by rubella ($391.60) and cytomegalovirus ($335.48); syphilis ($203.06) and toxoplasmosis ($171.42) had the lowest despite high frequency (S. Table 1; S. Figure 2). Regionally, the South consistently had the highest costs, while other regions showed lower and overlapping ranges. Nationally, costs increased 2008–2011, declined 2012–2016, and stabilized thereafter. All regions showed a significant decreasing trend in mean costs (*P* < 0.002).

### Hospitalization rates

**Figure 3.**
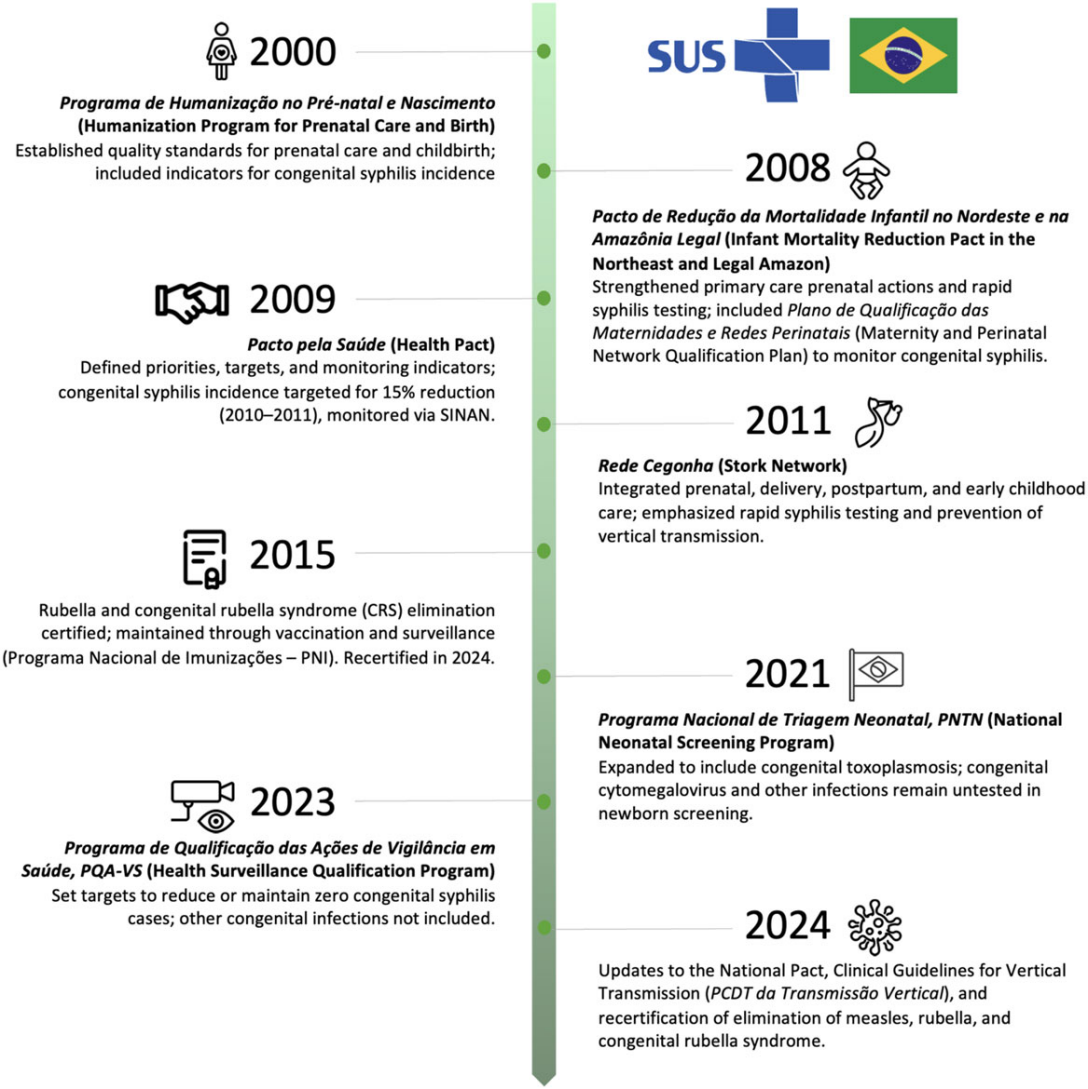
Major milestones in the Brazilian Unified Health System (SUS) response to congenital infections, 2000–2024.

From 2008 to 2024, hospitalization rates rose in all regions, with widening disparities. In 2008, rates were highest in the Northeast, followed by North, Southeast, Central-West, and South. By 2024, the Southeast led, followed by Northeast, North, Central-West, and South. After 2012, the Southeast consistently had the highest rates and Central-West the lowest. Congenital syphilis dominated national trends, while toxoplasmosis and cytomegalovirus rose steadily and herpes increased later. Rubella declined after 2010 and remained low (Figure 2; S. Table 2; S. Figure 3).

In the Southeast, syphilis rates increased from less than two per 1,000 in 2008 to more than twelve per 1,000 by 2021. All regions were impacted, with lower but increasing rates in the Central-West and South. By 2024, toxoplasmosis exceeded 1.2 per 1,000 in the Southeast and Northeast, while herpes rates rose after 2018, particularly in the Northeast and South. Cytomegalovirus increased mainly after 2015, especially in the Southeast. Since 2020, rubella remained low.

Statistical analysis revealed significant increases for all infections except rubella (non-significant decrease, *P* = 0.48). Increasing hospitalization trends were also significant in South (*P* < 0.001), Southeast (*P* = 0.01), and Northeast (*P* = 0.01), but not in North (*P* = 0.1) or Central-West (*P* = 0.1).

### Length of stay and ICU use

Mean length of stay (LOS) declined slightly or remained stable for most infections between 2008 and 2024. LOS also decreased for rubella (7 to 5.7 d), toxoplasmosis (6.4 to 4.7), and cytomegalovirus (10.2 to 6.2). Herpes showed a slight recent decrease (9.6 to 9.4 d), with a notable 2010 peak of 16.3 days (S. Table 3).

A one-way ANOVA confirmed significant LOS variation across infections (*F*(4,20) = 8.2, *P* = 0.01). Congenital herpes, cytomegalovirus, and syphilis had the longest stays, toxoplasmosis the shortest, and rubella intermediate. National mean LOS decreased marginally over time, with significant declines only the Southeast and Northeast (*P* < 0.05).

Regarding Intensive Care Unit (ICU) use, herpes showed the highest and most variable rates, while cytomegalovirus declined over time. Rubella fluctuated before declining, and syphilis and toxoplasmosis remained low and stable (<6%), with slight 2024 increases (S. Figure 4; S. Table 4). Regionally, the South recorded the highest ICU use, the Northeast the lowest, and other regions intermediate (S. Figure 5).

### In-hospital mortality rate

In-hospital mortality rates were lower for congenital syphilis and toxoplasmosis than for rubella, herpes, and cytomegalovirus. Mortality decreased or stabilized over time for all infections, declining nationally from 0.73 in 2008 to 0.13 in 2024 (S. Figure 6; S. Table 5). Regionally, mortality was lower in the South and Southeast, and higher in the North, Central-West, and Northeast.

### Hospitalizations outside the municipality of residence and mean distances

A considerable share of hospitalizations (17%–30% annually) occurred outside patients’ municipalities, increasing nationally from 26.7% in 2008 to 29.5% in 2024. The Northeast consistently had the highest proportion (∼40%), while the South showed the steepest rise (20.6% to 34.3%).

The median travel distances decreased in the Southeast (50.4 to 33.5 km), North (88.5 to 84.5 km), and nationally (61.8 to 59.0 km) between 2008 and 2024, while they stayed constant in the South (∼40 km). In the Northeast (63.2 to 66.6 km) and especially in the Central-West (73.3 to 98.8 km), distances grew. Distances decreased for syphilis (59.7 to 55.5 km), herpes (35.4 to 27.8 km), and rubella (67.3 to 63.4 km), while they increased for toxoplasmosis (71.4 to 77.9 km) and cytomegalovirus (60.0 to 64.7 km) (S. Figure 7; S. Table 6).

In 2008, the South and Southeast were characterized by shorter travel distances, whereas the North showed the longest distances and the lowest proportion of flows within 50 km. By 2024, the Northeast remained intermediate, the South and Southeast continued to exhibit shorter flows, and the Central-West resembled the North. Patients who traveled more than 1,500 km from the North and Northeast were admitted to three Central-West hospitals in 2024. Herpes had the shortest flows in both years, while toxoplasmosis consistently had the longest distances and the largest percentage of flows over 100 km (Table 2).

**Table 2.** Distances traveled in hospitalizations for congenital infections outside the patient’s municipality of residence

Distance, in kilometers (2008)	Min.^(a)^	Max.	Mean	Median ^(d)^	% Patient flows <50km ^(c)^	% Patient flows >100km ^(c)^
**Brazil**	**2.3**	**940.8**	**30.6**	**61.8**	**63%**	**16%**
North	10.30	940.76	51.45	88.5	48%	27%
Northeast	2.28	784.59	36.44	63.2	60%	15%
South	4.2	158.4	23.1	40.2	68%	8%
Southeast	2.9	882.8	20.8	50.4	80%	12%
Middle West	5.3	287.3	43.0	73.3	59%	22%
C. Syphilis	2.3	940.8	28.8	59.7	66%	13%
C. Toxoplasmosis	3.8	350.8	32.9	71.4	59%	29%
C. Herpes	2.9	118.1	23.3	35.4	79%	7%
C. Rubella	2.9	784.6	41.1	67.3	56%	19%
C. Cytomegalovirus	14.2	333.4	35.4	60.0	67%	17%
**Distance, in kilometers (2024)**	**Min.** ^**(a)**^	**Max.**	**Mean**	**Median** ^**(d)**^	**% Patient flows <50km** ^**(c)**^	**% Patient flows >100km** ^**(c)**^
**Brazil**	**1.80**	**831.8**	**31.5**	**59.0**	**65%**	**18%**
North	3.5	701.1	58.4	84.5	44%	31%
Northeast	3.6	793.0	41.2	66.6	59%	23%
South	2.2	785.5	20.6	40.1	82%	6%
Southeast	1.8	407.8	23.4	33.6	83%	5%
Middle West	5.3	1.941.9	63.9	98.9	42%	35%
C. Syphilis	1.8	793.0	30.1	55.5	67%	14%
C. Toxoplasmosis	3.4	831.8	47.2	77.9	51%	25%
C. Herpes	3.4	112.0	20.6	27.8	83%	3%
C. Rubella	3.3	628.7	31.3	64.0	63%	23%
C. Cytomegalovirus	3.4	520.7	38.0	64.7	62%	19%

*Source*: Developed by authors, with data extracted from SIH/SUS, considering the selected ICD-10 codes.

(a) Municipal boundaries often follow sociopolitical and geographic factors rather than physical distance. In many cases, especially in densely populated areas, municipalities may be directly adjacent, separated only by a street, avenue, or river, resulting in very low distances. For example, the municipalities of Teresina (Piauí) and Timon (Maranhão), located in different Northeastern states, are separated by less than 6 km; (b) Considering the total number of hospitalizations; (c) Considering the total number of hospitalizations with patient flows (that is, those that took place outside the patients’ municipality of residency); (d) Given the high standard deviation (SD), the median is pertinent measure in addition to the mean values, as it represents the central value of the distribution and mitigates the influence of extreme values that may distort the mean. Abbreviation: C, congenital.

## Discussion

This nationwide analysis provides a comprehensive overview of the hospital burden of congenital infections in Brazil over nearly two decades. The study reveals a steady rise in hospitalizations, largely driven by congenital syphilis, which accounted for most cases and shaped national and regional trends. Other infections, such as toxoplasmosis and cytomegalovirus, showed smaller progressive increases, while herpes surged later, and rubella remained low or stable. Despite regional variation, the data highlight persistent disparities in hospitalization rates, costs, intensive care use, and in-hospital mortality, underscoring that congenital infections remain a major public health challenge.

The rising costs and hospitalization burden reflect persistent weaknesses in prenatal care and in the integration of maternal–child health services within SUS. In syphilis, late detection and recurrent infections expose failures in partner screening and treatment adherence, perpetuating transmission despite effective, low-cost therapy. Strengthening universal screening, ensuring timely treatment, and expanding secondary prevention, through health education and vaccination, are essential to reduce incidence and severity. The regional disparities observed reflect epidemiological differences and structural inequities in healthcare delivery, emphasizing the need for region-specific policies to address local vulnerabilities and resource gaps.

### Correlation with existing literature

Our findings of a marked rise in hospitalizations for congenital syphilis, with strong regional variation, are consistent with the broader literature documenting a sustained and spatially expanding epidemic of congenital syphilis across Brazil. Multiple spatiotemporal analyses have shown steep increases in incidence, particularly in the South, Southeast, and Central-West regions, and the spread from coastal urban centers into inland municipalities characterized by social vulnerability and incomplete prenatal care.^2,3,7,15–19^ These studies corroborate our observation that congenital syphilis is the main driver of hospitalization trends and disproportionately affects regions with weaker maternal health infrastructure. By contrast, the lack of robust surveillance data for other congenital infections in the literature parallels our finding that their hospital burden, although relevant, is less well characterized and receives limited public health attention.

The high volume of hospitalizations, prolonged stays, and increased costs documented in our study resonate with evidence from LMICs showing that congenital infections impose disproportionate clinical and economic burdens compared with high-income countries. Paixão et al,^8^ demonstrated that children with congenital syphilis in Brazil faced a six-fold higher risk of hospitalization, particularly in the neonatal period, aligning with our finding of syphilis as the predominant cause of infant admissions. Similarly, the elevated resource demands we observed for cytomegalovirus and herpes mirror global reports of higher birth prevalence of congenital cytomegalovirus in LMICs and longer, costlier hospitalizations for neonatal herpes.^20–23^ Although rubella hospitalizations declined in our analysis, evidence from China indicates that congenital rubella syndrome still carries the highest individual economic burden among TORCH infections.^22^ These parallels reinforce that congenital infections, especially syphilis and cytomegalovirus, remain major drivers of pediatric hospital resource utilization in resource-limited settings.

In 2008, SUS implemented a nationwide rubella vaccination campaign that immunized 67.9 million individuals, achieving 96.7% coverage of the target population of adults aged 20–39 years, with additional inclusion of adolescents aged 12–19 years in selected states. Following five consecutive years without documented endemic transmission, Brazil was officially certified as having eliminated rubella in 2015.^24^

The persistent rise in congenital syphilis hospitalizations underscores limitations of Brazil’s prevention strategies despite national initiatives (Figure 3). Interrupted time series analyses show some reductions in high-burden municipalities after targeted interventions, but overall incidence remains high compared with high-income countries.^25^ Regional differences in hospitalization rates align with evidence of unequal primary care performance, especially in partner management and health education.^5^ Although national treatment coverage exceeds 80%, congenital syphilis has continued to rise, diverging from the declining trajectories of HIV and hepatitis B^26^. The pandemic’s disruption of prenatal services, reflected in delayed diagnoses, may also have contributed to the hospital burden observed.^27^ For toxoplasmosis, steady hospitalization growth is consistent with literature showing Brazil’s higher burden than high-income countries, driven by environmental risk factors, late maternal diagnoses, and absent systematic prenatal screening.^28,29^

In addition to true increases in disease burden, improvements in diagnostic capacity and test utilization may have contributed to the rising number of hospitalizations observed over time. Greater awareness and improved adherence to diagnostic protocols may have reduced underdiagnosis. As a result, part of the observed increase in hospital admissions may reflect enhanced case detection rather than a proportional rise in incidence.

An important aspect of our findings concerns Brazil’s persistent regional inequalities shaping the burden of congenital infections. The North and Northeast, marked by lower socioeconomic indicators, limited health infrastructure, and poorer prenatal care, consistently showed higher hospitalization rates due to structural barriers such as fewer specialized maternity services, lower syphilis and toxoplasmosis screening coverage, and limited laboratory capacity. In contrast, the more developed South and Southeast, with greater availability of tertiary hospitals, exhibited lower hospitalization rates but higher costs and ICU use, reflecting more advanced case management and broader access to intensive care. This geographic barrier to accessing healthcare in the North and Northeast has already been identified in previous studies.^30,31^

### Limitations and strengths

Several limitations must be considered. First, the retrospective, population-based time series design has cohort-like features but is not a true cohort, as it analyzes population-level hospital records rather than individual follow-up; nonetheless, the 17-year span and repeated measures permit meaningful assessment of temporal trends. Second, reliance on DATASUS SIH, a robust yet limited secondary database dependent on ICD-10 coding accuracy, introduces risks of underreporting, misclassification, and regional variability, especially for less frequent congenital infections that constitute a small proportion of infant hospitalizations and may not appear among leading causes of admission. Moreover, changes over time in surveillance practices, diagnostic capacity, and reporting quality may have contributed to apparent increases in hospitalization rates, reflecting improved case detection rather than true changes in disease incidence. Individual clinical outcomes, such as neurological sequelae, hearing loss, congenital malformations, cannot be assessed because the database provides only hospitalization-related proxies. Third, regional differences in healthcare infrastructure and resource availability may influence observed patterns, including higher ICU utilization in more economically developed regions, which may reflect greater access, heightened clinical awareness, or lower thresholds for admission rather than differences in disease severity alone; these factors may also partially explain regional disparities in mortality. Fourth, it was beyond the scope of this study to analyze the incidence of the infections among the general population (all ages), which limits conclusions on transmissibility. Finally, causality between public health interventions and observed trends cannot be established.

Despite these limitations, the study has notable strengths. It is the first nationwide, long-term analysis of the hospital burden of multiple congenital infections in Brazil, integrating data on hospitalization rates, costs, length of stay, ICU use, mortality, and regional disparities. Standardized, publicly accessible data support transparency and reproducibility, and the extended observation period captures temporal patterns missed by shorter studies. By analyzing infections collectively, the study offers a comprehensive perspective critical for policy planning, resource allocation, and strengthening maternal–child health networks.

From a public health perspective, these findings offer actionable insights for Brazil’s Unified Health System. Nationwide analyses based on DATASUS data are routinely used by health authorities to inform planning and resource allocation. By quantifying hospitalizations that are largely preventable through effective prenatal screening and timely treatment, our results highlight opportunities for cost avoidance and efficiency gains. The pronounced regional disparities in mortality further support the need for targeted investments in prenatal care and diagnostic capacity, particularly in the North and Northeast.

## Conclusions

From 2008 to 2024, congenital syphilis accounted for nearly 90% of hospitalizations in Brazil; toxoplasmosis, cytomegalovirus, and herpes also increased, while rubella decreased. This nationwide, population-based study reveals a nearly fourfold increase in hospitalizations for congenital infections in Brazil. These results demonstrate the significant, long-lasting effects of congenital infections on the Unified Health System as well as the regional differences in hospitalization rates, expenses, ICU utilization, and mortality. As congenital infections continue to be preventable causes of infant morbidity and mortality, public health policies should prioritize region-specific measures to expand prenatal care, strengthen surveillance beyond syphilis, and integrate maternal–child health services. Ensuring equitable access to prenatal screening, treatment, and vaccination is essential to reduce hospitalizations, relieve economic pressures, and promote equity in maternal and neonatal care.

## Supporting information

10.1017/ash.2026.10300.sm001 Callado et al. supplementary material Callado et al. supplementary material

## Data Availability

All data used in this study were obtained from publicly available, de-identified datasets provided by the Brazilian Unified Health System (Sistema Único de Saúde, SUS) through official platforms, such as the Hospital Information System (SIH). These datasets include aggregated hospitalization records and do not contain individual patient identifiers. The study protocol, statistical analysis plan, and the data extraction procedures are available from the corresponding author upon reasonable request. As the primary data are publicly accessible, no additional approval is required for their use. Access to the raw SUS datasets can be obtained via the official TABWIN software platform (http://tabnet.datasus.gov.br) or the DATASUS repository. Researchers seeking to reproduce or extend the analyses can do so using these publicly available resources.

## Supplementary material

The supplementary material for this article can be found at https://doi.org/10.1017/ash.2026.10300
