# Supplementary material for: Hospitalizations for congenital infections in Brazil’s unified health system: nationwide trends and regional disparities, 2008–2024
Source: Antimicrob Steward Healthc Epidemiol. 2026 Feb 3;6(1):e40. doi: 10.1017/ash.2026.10300 (PMC12877911; doi:10.1017/ash.2026.10300)
Supplement: Callado et al. supplementary material [file S2732494X26103003sup001.docx]

**SUPPLEMENTARY MATERIAL**

**Hospitalizations for Congenital Infections in Brazil’s Unified Health System:**

**Nationwide Trends and Regional Disparities, 2008–2024**

Callado, Siqueira, Corrêa, Delpino, Marra, Santana

**TABLE OF CONTENTS:**

**SUPPLEMENTARY FIGURES**

**S. Figure 1. Number of hospitalizations, per year and congenital infection ………………………………………………………………………….. 4**

**S. Figure 2. Mean SUS reimbursement (in US$), per congenital infection and year ………………………………………………………….……... 7**

**S. Figure 3. Hospitalization rates for the five congenital infections (2008 vs. 2016 vs. 2024) ……………………………………………………...… 8**

**S. Figure 4. Proportion of hospitalizations with ICU use, per year and congenital infection …………..………….…………..…………..………... 9**

**S. Figure 5. Proportion of hospitalizations for the selected congenital infections with ICU use, per year and region …………..…………..…….. 10**

**S. Figure 6. In-hospital mortality rates, per congenital infection and year …………………..…………..…………………..………..…………..….. 11**

**S. Figure 7. Percentage of hospitalizations for congenital infections outside patients’ municipality of residence, per year and region ………….. 12**

**SUPPLEMENTARY TABLES**

**S. Table 1. Mean SUS cost per hospitalization (2008–2024) …………………………………………………………………………………….. 13**

**S. Table 2. Hospitalization rates (2008–2024) ………………………………………………………………………………….……………….….. 15**

**S. Table 3. Mean in-hospital Length of Stay (LOS), in days (2008–2024) …………………………………………………………………….. 17**

**S. Table 4. Percentage of hospitalizations with Intensive Care Unit (ICU) use (2008–2024) ………………………………………...…….. 19**

**S. Table 5. In-hospital mortality rate (2008–2024) ………………………………………………………………………………………………... 21**

**S. Table 6. Percentage of Hospitalizations Outside Patients’ Municipality of Residence (2008–2024) ………………………………….... 23**

**SUPPLEMENTARY MATERIAL – STATISTICAL ANALYSES**

**1. Differences Between Regions in 2024 …………………………………………………………………………………………………………… 25**

**1.1. Hospitalization Rate …………………………………………………………...………………………………………………………………… 25**

**1.2. Mean Hospitalization Cost ……………………….…………………………...………………………………………………………………… 25**

**1.3. Mean Length of Stay …………………………………………………………...………………………………………………………………… 26**

**2. Temporal Trends (2008–2024) …………………..……………………………...………………………………………………………………… 26**

**2.1. Hospitalization Rate by Congenital Infection ………...……………………………………………………………………………………… 26**

**2.2. Hospitalization Rate by Region ………...………………………………………………………………………………….…………………… 27**

**2.3. Mean Hospitalization Cost by Region ………...…………………………………………………..…………………………………………… 27**

**2.4. Mean Length of Stay by Region ………...……………………………………………………………………………………………………… 27**

**3. Differences Between Congenital Infections in 2024 ………...…………….…………………………………………………………………… 28**

**3.1. Hospitalization Rate 3.2. Mean Hospitalization Cost ………...…………..………………………………………………………………… 28**

**3.3. Mean Length of Stay ………...…………………………………………………………………………………………………………………… 29**

**4. In-Hospital Mortality by Region (2008–2024) ………...…………………………………...…………………………………………………… 29**

**4.1. Differences Between Regions ………...……………………………………………………………..…………………………………………… 29**

**4.2. Temporal Evolution of Mortality ………...…………………………………………………………..………………………………………… 30**

**S. Figure 1.** Number of hospitalizations, per year and congenital infection


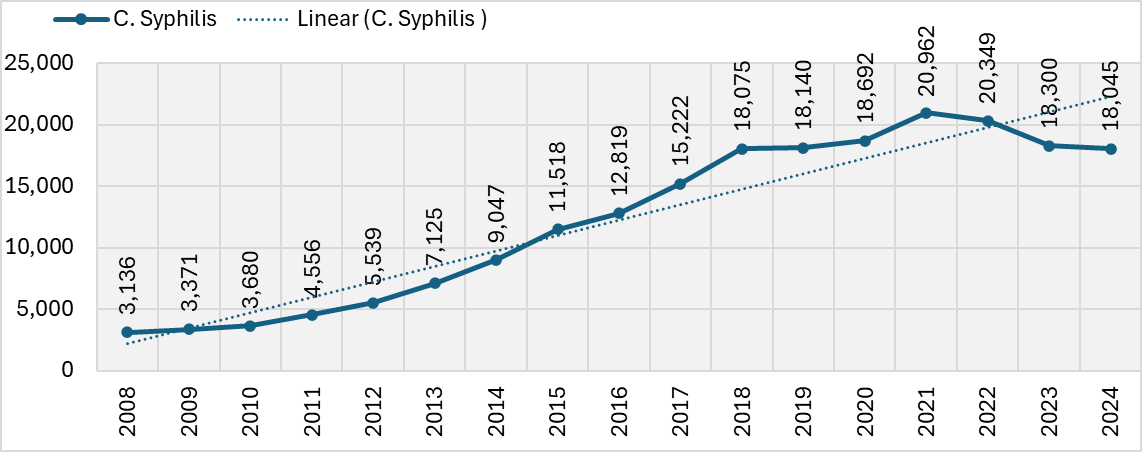


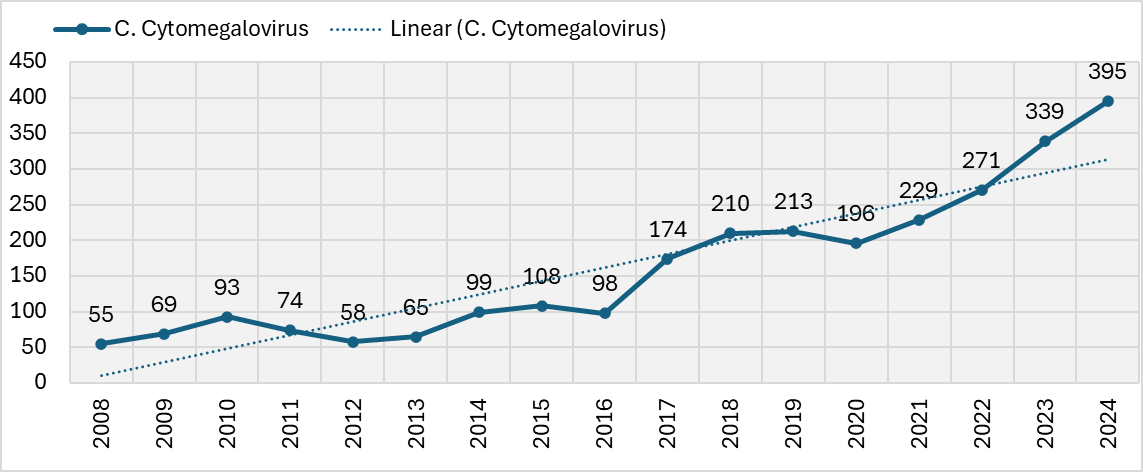


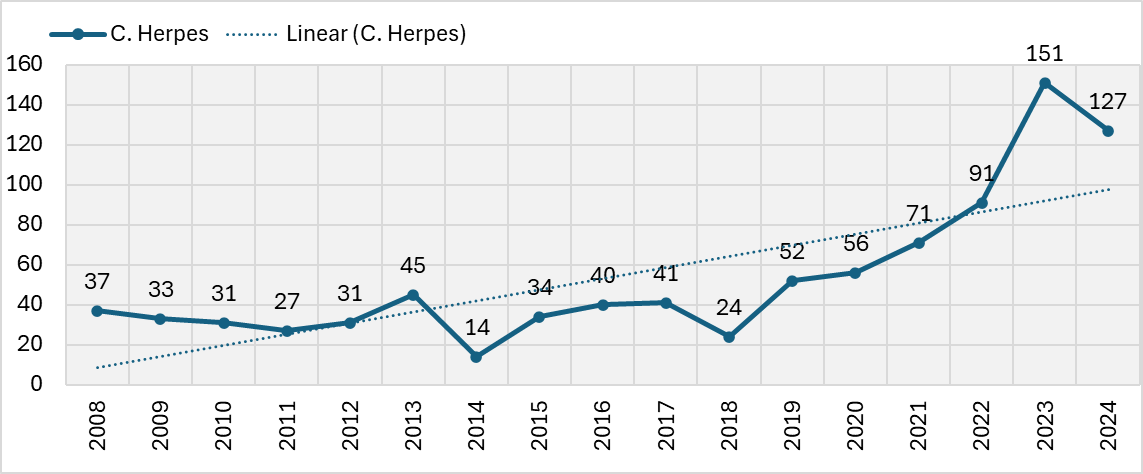


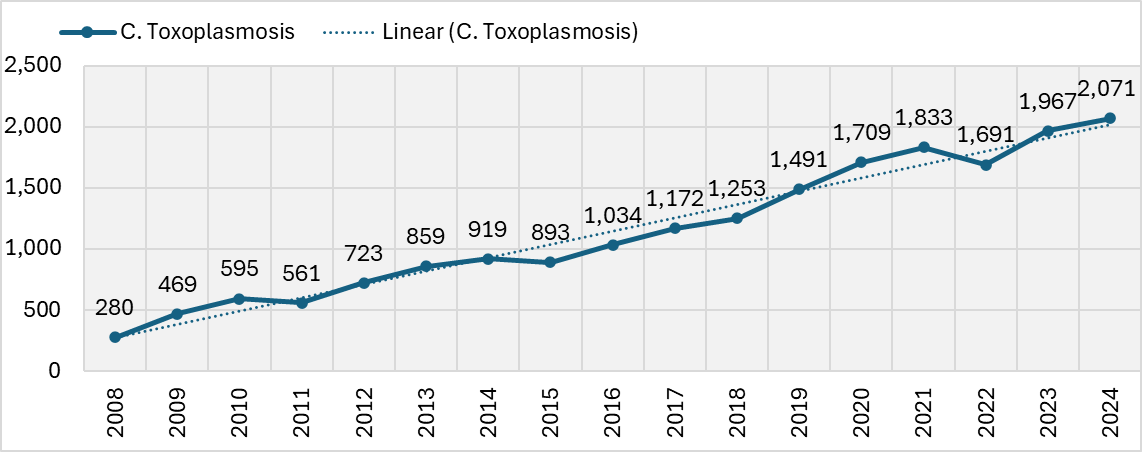


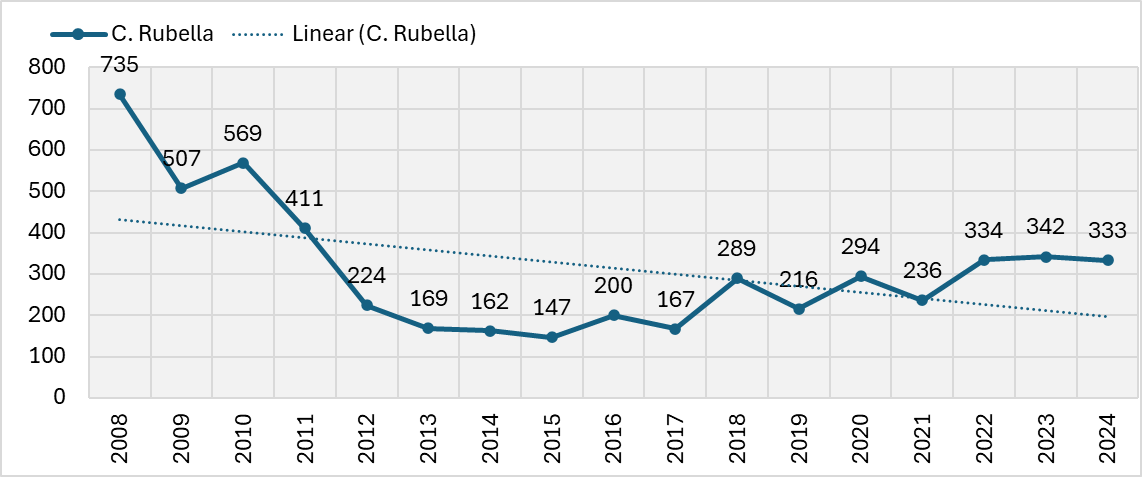


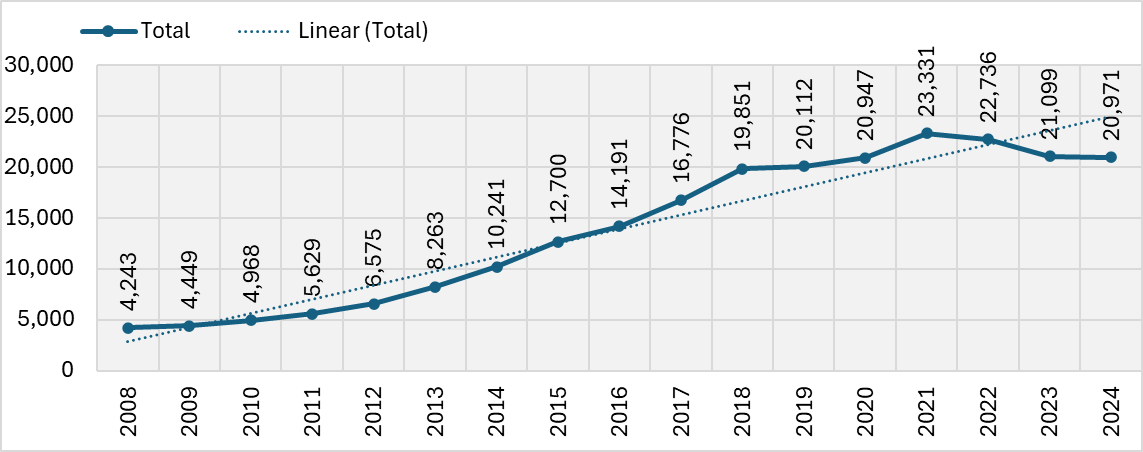


***Source****: Developed by authors, with data extracted from SIH/SUS, considering the selected ICD-10 codes.*

**S. Figure 2.** Mean SUS' reimbursement (in US$), per congenital infection and year


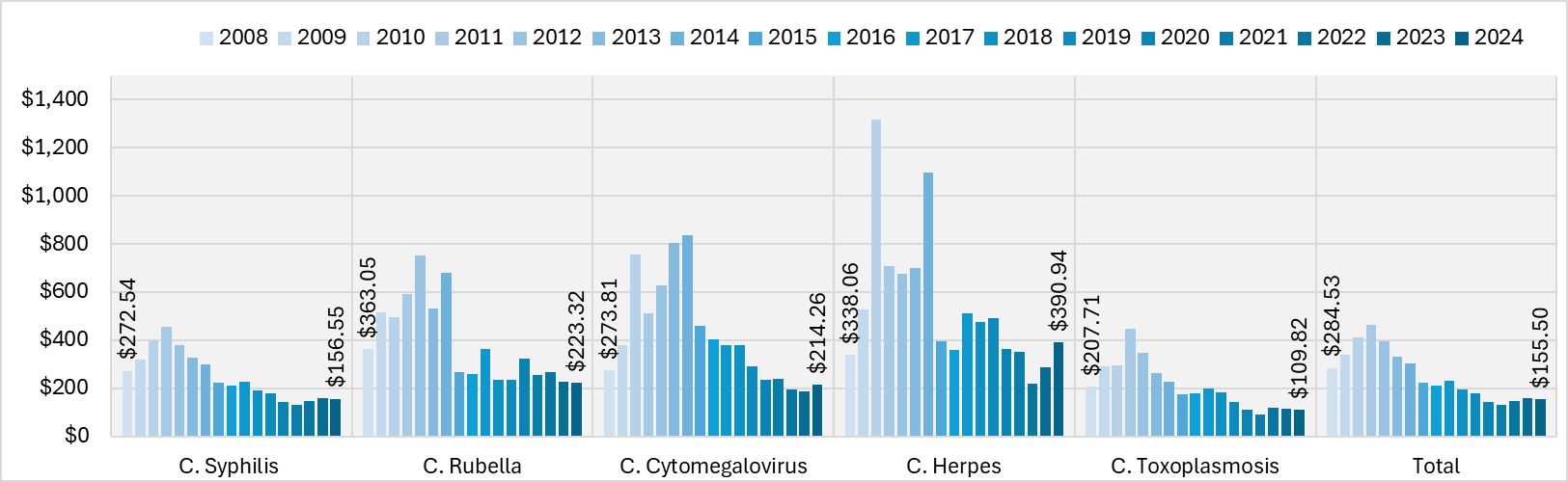


***Source****: Developed by authors, with data extracted from SIH/SUS, considering the selected ICD-10 codes.*

**S. Figure 3.** Hospitalization rates for the five congenital infections (2008 vs. 2016 vs. 2024)


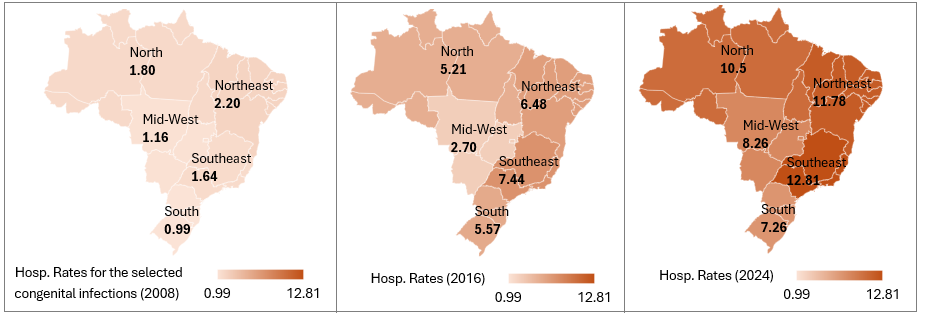


***Source****: Developed by authors, with data extracted from SIH/SUS, considering the selected ICD-10 codes.*

*** Hospitalizations per 1,000 inhabitants with up to 1 year old and no coverage by private health insurance*

**S. Figure 4**. Proportion of hospitalizations with ICU use, per year and congenital infection


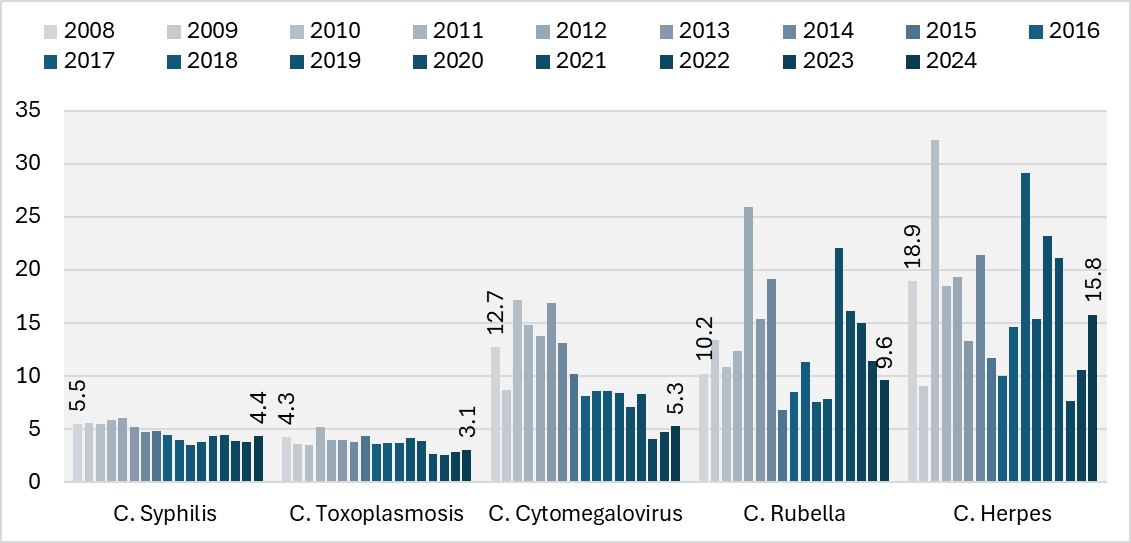


***Source****: Developed by authors, with data extracted from SIH/SUS, considering the selected ICD-10 codes.*

**S. Figure 5.** Proportion of hospitalizations for the selected congenital infections with ICU use, per year and region


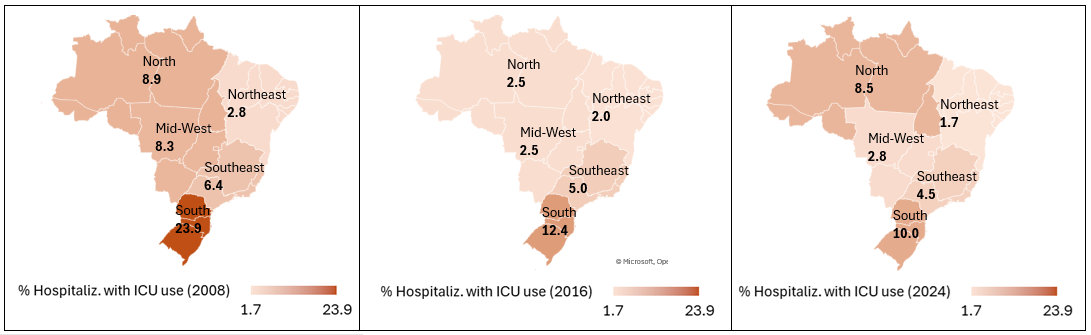


***Source****: Developed by authors, with data extracted from SIH/SUS, considering the selected ICD-10 codes.*

**S. Figure 6.** In-hospital mortality rates, per congenital infection and year


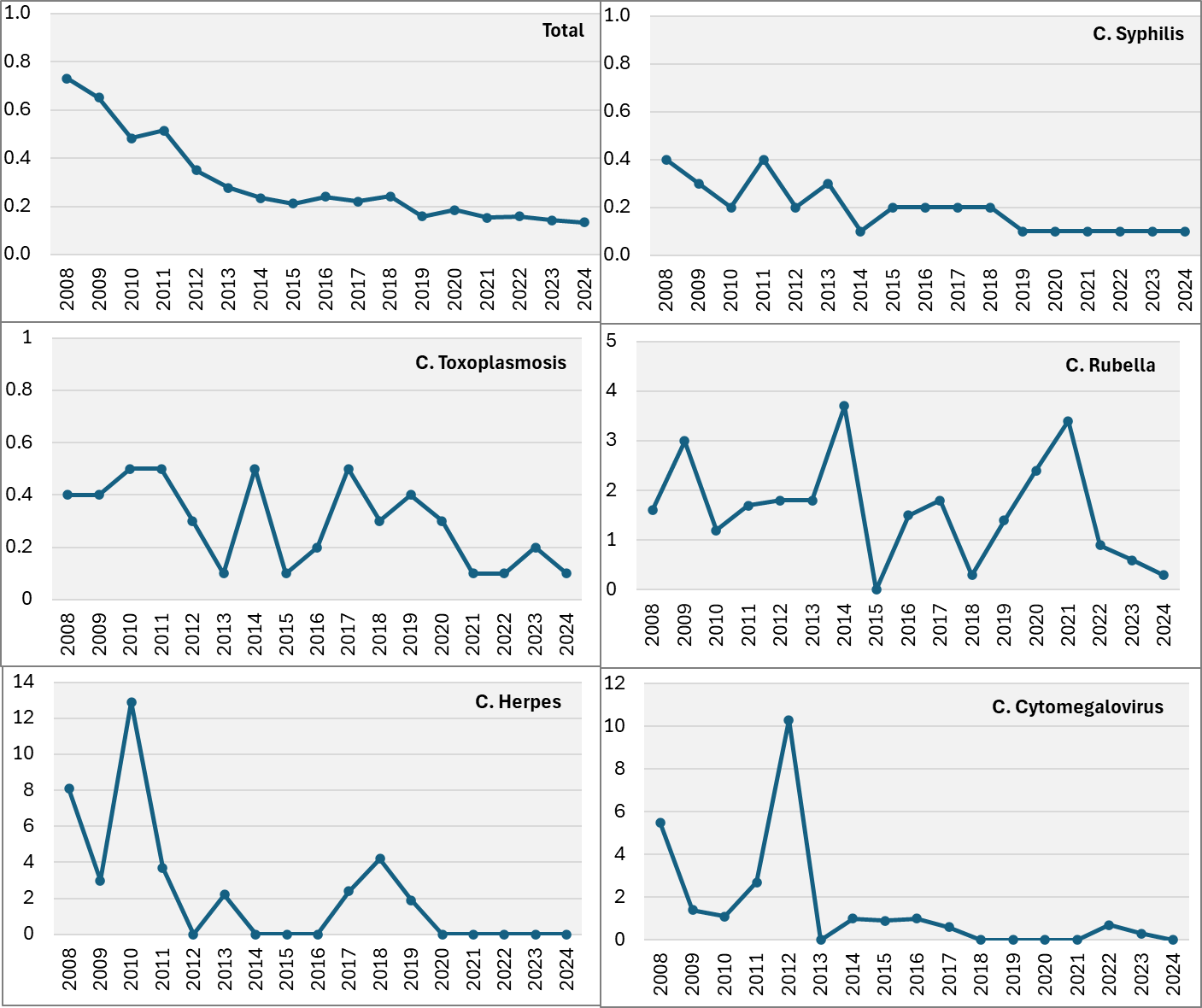


***Source****: Developed by authors, with data extracted from SIH/SUS, considering the selected ICD-10 codes. The rates represent the number of in-hospital deaths by 1,000 hospitalizations***S. Figure 7.** Percentage of hospitalizations for congenital infections outside patients’ municipality of residence, per year and region


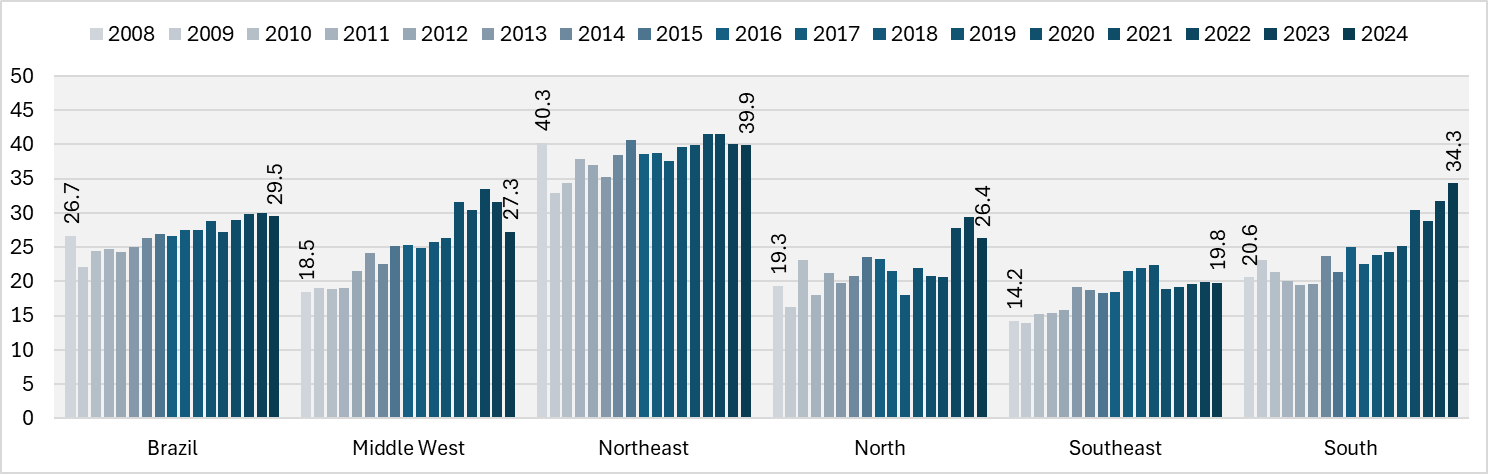


***Source****: Developed by authors, with data extracted from SIH/SUS, considering the selected ICD-10 codes.*

**S. Table 1.** Mean SUS' cost per hospitalization

|  |  |  |  |  | **(2008-2024)** | | | | |
| --- | --- | --- | --- | --- | --- | --- | --- | --- | --- |
| **Infection** | **Region** | **2008** | **2016** | **2024** | **Min** | **Max** | **Mean** | **Median** | **SD** |
| Congenital Syphilis | **Brazil** | $ 272.54 | $ 209.90 | $ 156.55 | $ 133.30 | $ 454.30 | $ 248.79 | $ 224.65 | $ 99.45 |
|  | Central-West | $ 238.49 | $ 153.73 | $ 133.68 | $ 91.32 | $ 427.17 | $ 198.35 | $ 153.73 | $ 102.96 |
|  | Northeast | $ 254.09 | $ 186.61 | $ 110.70 | $ 105.53 | $ 428.18 | $ 219.05 | $ 186.95 | $ 102.51 |
|  | North | $ 308.90 | $ 206.83 | $ 219.93 | $ 136.65 | $ 463.48 | $ 275.34 | $ 242.09 | $ 97.73 |
|  | Southeast | $ 203.54 | $ 196.26 | $ 157.33 | $ 127.30 | $ 335.14 | $ 206.42 | $ 203.54 | $ 60.16 |
|  | South | $ 690.35 | $ 345.71 | $ 239.97 | $ 232.60 | $ 1,262.70 | $ 538.78 | $ 345.71 | $ 343.37 |
| Congenital Rubella | **Brazil** | $ 363.05 | $ 259.76 | $ 223.32 | $ 223.32 | $ 752.95 | $ 387.50 | $ 325.25 | $ 172.92 |
|  | Central-West | $ 1,003.41 | $ 248.47 | $ 282.37 | $ 105.17 | $ 1,169.82 | $ 522.86 | $ 425.95 | $ 329.82 |
|  | Northeast | $ 227.74 | $ 255.00 | $ 137.34 | $ 131.45 | $ 763.49 | $ 305.71 | $ 276.35 | $ 157.95 |
|  | North | $ 357.20 | $ 72.79 | $ 325.84 | $ 43.18 | $ 2,070.44 | $ 393.76 | $ 220.70 | $ 553.09 |
|  | Southeast | $ 458.05 | $ 293.66 | $ 209.67 | $ 179.94 | $ 1,033.85 | $ 399.91 | $ 301.57 | $ 240.20 |
|  | South | $ 596.26 | $ 141.25 | $ 758.87 | $ 131.72 | $ 3,474.30 | $ 842.33 | $ 719.20 | $ 771.73 |
| Congenital Cytomegalovirus | **Brazil** | $ 273.81 | $ 404.30 | $ 214.26 | $ 187.01 | $ 838.53 | $ 422.65 | $ 380.29 | $ 215.77 |
|  | Central-West | $ 111.47 | $ 82.93 | $ 107.91 | $ 64.60 | $ 1,723.78 | $ 407.74 | $ 173.24 | $ 469.30 |
|  | Northeast | $ 253.18 | $ 144.34 | $ 126.79 | $ 79.07 | $ 927.23 | $ 282.81 | $ 215.84 | $ 215.86 |
|  | North | $ 622.00 | $ 2,365.60 | $ 56.29 | $ 56.29 | $ 2,365.60 | $ 440.57 | $ 263.34 | $ 544.21 |
|  | Southeast | $ 254.99 | $ 398.96 | $ 281.24 | $ 136.36 | $ 959.58 | $ 464.91 | $ 398.96 | $ 251.01 |
|  | South | $ 295.36 | $ 1,070.14 | $ 517.92 | $ 89.85 | $ 2,361.20 | $ 896.29 | $ 849.91 | $ 685.79 |
| Congenital Herpes | **Brazil** | $ 338.06 | $ 358.69 | $ 390.94 | $ 221.29 | $ 1,315.95 | $ 542.06 | $ 474.47 | $ 289.28 |
|  | Central-West | $ 129.01 | $ 82.80 | $ 85.93 | - | - | - | - | - |
|  | Northeast | $ 472.97 | $ 178.71 | $ 201.11 | $ 82.06 | $ 1,789.87 | $ 481.60 | $ 431.50 | $ 426.80 |
|  | North | $ 172.60 | $ 786.50 | $ 130.45 | - | - | - | - | - |
|  | Southeast | $ 289.04 | $ 745.64 | $ 363.05 | $ 139.19 | $ 1,121.30 | $ 581.09 | $ 558.73 | $ 288.00 |
|  | South | $ 164.27 | $ 120.74 | $ 647.19 | $ 90.53 | $ 2,564.52 | $ 540.13 | $ 437.04 | $ 586.82 |
| Congenital Toxoplasmosis | **Brazil** | $ 207.71 | $ 179.66 | $ 109.82 | $ 91.82 | $ 447.49 | $ 206.58 | $ 183.92 | $ 96.91 |
|  | Central-West | $ 171.27 | $ 126.95 | $ 102.00 | $ 88.72 | $ 379.43 | $ 170.65 | $ 177.39 | $ 68.72 |
|  | Northeast | $ 206.61 | $ 141.78 | $ 96.62 | $ 73.09 | $ 730.58 | $ 203.85 | $ 141.78 | $ 160.00 |
|  | North | $ 516.22 | $ 123.83 | $ 97.05 | $ 97.05 | $ 516.22 | $ 182.00 | $ 157.62 | $ 100.71 |
|  | Southeast | $ 182.44 | $ 225.41 | $ 128.18 | $ 100.69 | $ 609.42 | $ 229.54 | $ 223.25 | $ 123.62 |
|  | South | $ 310.10 | $ 167.24 | $ 118.96 | $ 97.25 | $ 1,140.50 | $ 325.05 | $ 228.75 | $ 277.73 |
| Total congenital infections | **Brazil** | $ 284.53 | $ 210.16 | $ 155.50 | $ 132.99 | $ 465.57 | $ 253.53 | $ 224.09 | $ 104.54 |
|  | Central-West | $ 410.11 | $ 150.45 | $ 129.68 | $ 106.36 | $ 433.56 | $ 225.92 | $ 150.45 | $ 126.90 |
|  | Northeast | $ 248.39 | $ 185.65 | $ 109.95 | $ 103.74 | $ 418.05 | $ 222.79 | $ 185.73 | $ 106.72 |
|  | North | $ 316.50 | $ 205.92 | $ 215.96 | $ 135.95 | $ 433.97 | $ 263.96 | $ 238.05 | $ 88.22 |
|  | Southeast | $ 230.13 | $ 202.42 | $ 161.27 | $ 129.65 | $ 390.97 | $ 220.91 | $ 213.07 | $ 73.83 |
|  | South | $ 633.00 | $ 328.13 | $ 238.10 | $ 229.41 | $ 1,202.56 | $ 520.14 | $ 341.99 | $ 329.13 |

***Source****: Developed by authors, with data extracted from SIH/SUS, considering the selected ICD-10 codes.*

** Number of hospitalizations/total SUS reimbursement value*

**S. Table 2.** Hospitalization rates

|  |  |  |  |  | **(2008-2024)** | | | | |
| --- | --- | --- | --- | --- | --- | --- | --- | --- | --- |
| **Infection** | **Region** | **2008** | **2016** | **2024** | **Min** | **Max** | **Mean** | **Median** | **SD** |
| Congenital Syphilis | **Brazil** | 1.29 | 5.61 | 9.39 | 1.29 | 10.28 | 5.74 | 5.61 | 2.99 |
|  | Central-West | 0.59 | 2.23 | 6.87 | 0.59 | 6.87 | 3.03 | 2.44 | 1.86 |
|  | Northeast | 1.55 | 5.92 | 9.99 | 1.55 | 11.01 | 6.19 | 5.92 | 3.13 |
|  | North | 1.68 | 4.86 | 10.12 | 1.58 | 10.12 | 5.29 | 4.86 | 2.73 |
|  | Southeast | 1.24 | 6.71 | 10.80 | 1.24 | 12.54 | 6.71 | 6.71 | 3.58 |
|  | South | 0.67 | 4.89 | 6.20 | 0.67 | 7.93 | 4.58 | 4.89 | 2.50 |
| Congenital Rubella | **Brazil** | 0.30 | 0.09 | 0.17 | 0.06 | 0.30 | 0.14 | 0.13 | 0.05 |
|  | Central-West | 0.29 | 0.04 | 0.06 | 0.02 | 0.38 | 0.12 | 0.07 | 0.08 |
|  | Northeast | 0.55 | 0.15 | 0.08 | 0.08 | 0.55 | 0.20 | 0.14 | 0.11 |
|  | North | 0.06 | 0.02 | 0.02 | 0.00 | 0.06 | 0.02 | 0.01 | 0.01 |
|  | Southeast | 0.18 | 0.09 | 0.44 | 0.06 | 0.44 | 0.17 | 0.13 | 0.08 |
|  | South | 0.22 | 0.02 | 0.04 | 0.01 | 0.22 | 0.07 | 0.05 | 0.05 |
| Congenital Cytomegalovirus | **Brazil** | 0.02 | 0.04 | 0.21 | 0.02 | 0.21 | 0.08 | 0.05 | 0.04 |
|  | Central-West | 0.01 | 0.03 | 0.20 | 0.01 | 0.20 | 0.06 | 0.04 | 0.04 |
|  | Northeast | 0.03 | 0.05 | 0.23 | 0.01 | 0.26 | 0.08 | 0.05 | 0.06 |
|  | North | 0.01 | 0.01 | 0.01 | 0.00 | 0.03 | 0.01 | 0.01 | 0.01 |
|  | Southeast | 0.03 | 0.06 | 0.34 | 0.02 | 0.34 | 0.12 | 0.09 | 0.07 |
|  | South | 0.01 | 0.03 | 0.04 | 0.01 | 0.07 | 0.03 | 0.03 | 0.01 |
| Congenital Herpes | **Brazil** | 0.02 | 0.04 | 0.21 | 0.02 | 0.21 | 0.08 | 0.05 | 0.04 |
|  | Central-West | 0.01 | 0.03 | 0.20 | 0.01 | 0.20 | 0.06 | 0.04 | 0.04 |
|  | Northeast | 0.03 | 0.05 | 0.23 | 0.01 | 0.26 | 0.08 | 0.05 | 0.06 |
|  | North | 0.01 | 0.01 | 0.01 | 0.00 | 0.03 | 0.01 | 0.01 | 0.01 |
|  | Southeast | 0.03 | 0.06 | 0.34 | 0.02 | 0.34 | 0.12 | 0.09 | 0.07 |
|  | South | 0.01 | 0.03 | 0.04 | 0.01 | 0.07 | 0.03 | 0.03 | 0.01 |
| Congenital Toxoplasmosis | **Brazil** | 0.12 | 0.45 | 1.08 | 0.12 | 1.08 | 0.54 | 0.45 | 0.25 |
|  | Central-West | 0.26 | 0.40 | 1.13 | 0.26 | 1.13 | 0.56 | 0.54 | 0.15 |
|  | Northeast | 0.06 | 0.32 | 1.42 | 0.04 | 1.42 | 0.49 | 0.32 | 0.36 |
|  | North | 0.04 | 0.32 | 0.34 | 0.04 | 0.97 | 0.43 | 0.36 | 0.16 |
|  | Southeast | 0.19 | 0.57 | 1.14 | 0.19 | 1.24 | 0.65 | 0.57 | 0.31 |
|  | South | 0.06 | 0.63 | 0.84 | 0.06 | 0.85 | 0.50 | 0.61 | 0.27 |
| Total congenital infections | **Brazil** | 1.74 | 6.21 | 10.92 | 1.74 | 11.49 | 6.52 | 6.21 | 3.28 |
|  | Central-West | 1.16 | 2.70 | 8.26 | 1.16 | 8.26 | 3.78 | 3.02 | 1.97 |
|  | Northeast | 2.20 | 6.48 | 11.78 | 1.98 | 12.20 | 7.00 | 6.48 | 3.50 |
|  | North | 1.80 | 5.21 | 10.50 | 1.80 | 10.50 | 5.75 | 5.21 | 2.67 |
|  | Southeast | 1.64 | 7.44 | 12.81 | 1.64 | 14.18 | 7.67 | 7.44 | 4.03 |
|  | South | 0.99 | 5.57 | 7.26 | 0.99 | 8.71 | 5.21 | 5.57 | 2.77 |

***Source****: Developed by authors, with data from SIH/SUS (for the selected ICD-10 codes), the Brazilian Institute of Geography and Statistics – IBGE (Population Projections, 2024 revision); and the National Supplementary Health Agency – ANS (beneficiary consultation, December 2008 to December 2024)*

** Number of hospitalizations per 1,000 population up to 1 year old with no private health insurance plan*

**S. Table 3.** Mean in-hospital Length of Stay - LOS, in days

|  |  |  |  |  | **(2008-2024)** | | | | |
| --- | --- | --- | --- | --- | --- | --- | --- | --- | --- |
| **Infection** | **Region** | **2008** | **2016** | **2024** | **Min** | **Max** | **Mean** | **Median** | **SD** |
| Congenital Syphilis | **Brazil** | 10.0 | 9.4 | 9.0 | 8.9 | 10.1 | 9.5 | 9.4 | 0.4 |
|  | Central-West | 9.6 | 9.0 | 9.2 | 8.6 | 10.0 | 9.3 | 9.2 | 0.3 |
|  | Northeast | 9.8 | 9.5 | 9.1 | 8.9 | 10.0 | 9.5 | 9.6 | 0.3 |
|  | North | 9.9 | 9.2 | 9.0 | 8.9 | 10.3 | 9.5 | 9.5 | 0.3 |
|  | Southeast | 10.1 | 9.3 | 8.8 | 8.6 | 10.3 | 9.4 | 9.3 | 0.6 |
|  | South | 10.6 | 9.3 | 9.3 | 9.3 | 10.8 | 9.9 | 9.5 | 0.5 |
| Congenital Rubella | **Brazil** | 7.0 | 7.2 | 5.7 | 5.7 | 8.5 | 7.2 | 7.2 | 0.6 |
|  | Central-West | 9.4 | 4.8 | 6.9 | 4.8 | 14.0 | 8.3 | 8.1 | 2.0 |
|  | Northeast | 6.5 | 7.7 | 6.6 | 5.1 | 9.6 | 7.3 | 7.1 | 0.8 |
|  | North | 7.5 | 3.7 | 7.5 | 2.0 | 14.0 | 6.5 | 6.2 | 2.1 |
|  | Southeast | 7.7 | 7.1 | 5.3 | 5.3 | 7.8 | 7.0 | 7.1 | 0.6 |
|  | South | 7.3 | 5.2 | 8.6 | 4.7 | 18.7 | 8.0 | 7.4 | 1.8 |
| Congenital Cytomegalovirus | **Brazil** | 10.2 | 10.3 | 6.2 | 6.2 | 11.8 | 9.2 | 9.3 | 1.2 |
|  | Central-West | 4.0 | 4.6 | 5.1 | 4.0 | 14.2 | 7.1 | 6.4 | 2.5 |
|  | Northeast | 13.8 | 7.4 | 6.2 | 6.2 | 13.8 | 8.2 | 7.4 | 1.5 |
|  | North | 11.0 | 24.0 | 3.0 | 3.0 | 27.0 | 11.2 | 9.5 | 5.5 |
|  | Southeast | 7.5 | 11.2 | 6.2 | 6.2 | 13.9 | 9.7 | 9.3 | 1.8 |
|  | South | 4.7 | 17.1 | 10.6 | 3.8 | 22.7 | 12.8 | 12.8 | 4.0 |
| Congenital Herpes | **Brazil** | 9.6 | 8.2 | 9.4 | 7.6 | 16.3 | 9.5 | 9.3 | 1.0 |
|  | Central-West | 5.2 | 10.0 | 12.0 | 2.0 | 13.7 | 6.1 | 5.0 | 3.1 |
|  | Northeast | 12.9 | 5.5 | 7.1 | 5.3 | 15.5 | 8.9 | 8.6 | 2.0 |
|  | North | 7.5 | 14.0 | 9.0 | 3.0 | 30.0 | 10.7 | 10.5 | 3.9 |
|  | Southeast | 6.4 | 12.8 | 9.5 | 6.4 | 76.3 | 13.7 | 10.1 | 7.4 |
|  | South | 7.0 | 8.5 | 11.4 | 5.0 | 14.0 | 9.8 | 9.8 | 1.7 |
| Congenital Toxoplasmosis | **Brazil** | 6.4 | 5.5 | 4.7 | 4.7 | 6.4 | 5.4 | 5.4 | 0.4 |
|  | Central-West | 5.7 | 4.5 | 4.6 | 4.5 | 6.8 | 5.7 | 5.7 | 0.6 |
|  | Northeast | 8.1 | 5.9 | 5.2 | 5.1 | 8.8 | 6.4 | 6.1 | 0.9 |
|  | North | 9.0 | 5.0 | 5.4 | 3.5 | 9.0 | 5.1 | 5.0 | 1.0 |
|  | Southeast | 5.8 | 5.7 | 4.1 | 4.1 | 7.1 | 5.3 | 5.3 | 0.6 |
|  | South | 6.8 | 5.2 | 4.0 | 4.0 | 6.8 | 5.4 | 5.6 | 0.7 |
| Total congenital infections | **Brazil** | 9.2 | 9.0 | 8.5 | 8.4 | 9.5 | 9.0 | 9.1 | 0.3 |
|  | Central-West | 8.6 | 8.2 | 8.5 | 7.4 | 9.1 | 8.5 | 8.5 | 0.3 |
|  | Northeast | 9.0 | 9.3 | 8.5 | 8.4 | 9.8 | 9.2 | 9.3 | 0.3 |
|  | North | 9.8 | 8.9 | 8.9 | 8.1 | 9.8 | 9.0 | 8.9 | 0.4 |
|  | Southeast | 9.3 | 9.0 | 8.2 | 8.2 | 9.5 | 8.9 | 9.0 | 0.5 |
|  | South | 9.5 | 8.8 | 8.7 | 8.7 | 10.0 | 9.3 | 9.1 | 0.4 |

***Source****: Developed by authors, with data extracted from SIH/SUS, considering the selected ICD-10 codes.*

** Total LOS in days/total number of hospitalizations*

**S. Table 4.** Percentage of Hospitalizations with Intensive Care Unit - ICU use

|  |  |  |  |  | **(2008-2024)** | | | | |
| --- | --- | --- | --- | --- | --- | --- | --- | --- | --- |
| **Infection** | **Region** | **2008** | **2016** | **2024** | **Min** | **Max** | **Mean** | **Median** | **SD** |
| Congenital Syphilis | **Brazil** | 5.5 | 4.5 | 4.4 | 3.5 | 6.0 | 4.7 | 4.5 | 0.7 |
|  | Central-West | 5.8 | 2.3 | 2.9 | 1.1 | 6.1 | 2.9 | 2.7 | 1.3 |
|  | Northeast | 1.7 | 1.9 | 1.6 | 1.3 | 3.1 | 1.8 | 1.6 | 0.3 |
|  | North | 8.7 | 2.5 | 8.6 | 2.1 | 9.1 | 4.4 | 3.2 | 1.9 |
|  | Southeast | 4.8 | 4.9 | 4.3 | 3.1 | 5.8 | 4.3 | 4.3 | 0.6 |
|  | South | 26.5 | 13.3 | 9.7 | 9.4 | 37.9 | 18.7 | 16.7 | 7.3 |
| Congenital Rubella | **Brazil** | 10.2 | 8.5 | 9.6 | 6.8 | 25.9 | 13.2 | 11.4 | 4.1 |
|  | Central-West | 19.6 | 12.5 | 18.2 | 7.7 | 44.4 | 21.9 | 18.9 | 8.3 |
|  | Northeast | 4.9 | 5.4 | 6.1 | 2.8 | 22.9 | 7.4 | 5.4 | 4.0 |
|  | North | 10.0 | - | 16.7 | 9.1 | 50.0 | 27.2 | 16.7 | 18.3 |
|  | Southeast | 18.9 | 14.5 | 7.1 | 5.4 | 51.1 | 15.0 | 12.1 | 6.5 |
|  | South | 20.6 | - | 66.7 | 9.1 | 71.4 | 44.7 | 46.8 | 13.2 |
| Congenital Cytomegalovirus | **Brazil** | 12.7 | 8.2 | 5.3 | 4.1 | 17.2 | 10.1 | 8.6 | 3.3 |
|  | Central-West | - | - | - | 3.9 | 50.0 | 20.6 | 20.8 | 12.7 |
|  | Northeast | 4.2 | 2.8 | 2.1 | 1.3 | 15.0 | 5.5 | 2.9 | 3.9 |
|  | North | 33.3 | 33.3 | - | 25.0 | 50.0 | 36.2 | 33.3 | 7.9 |
|  | Southeast | 16.7 | 6.5 | 8.0 | 3.9 | 20.0 | 11.8 | 12.9 | 4.6 |
|  | South | 33.3 | 37.5 | 16.7 | 11.8 | 66.7 | 30.5 | 33.3 | 13.6 |
| Congenital Herpes | **Brazil** | 18.9 | 10.0 | 15.8 | 7.7 | 32.3 | 17.2 | 15.8 | 5.5 |
|  | Central-West | - | - | - | 16.7 | 16.7 | 16.7 | 16.7 | 0.0 |
|  | Northeast | 33.3 | 4.2 | 5.7 | 4.2 | 42.1 | 18.3 | 17.7 | 9.8 |
|  | North | - | - | - | 25.0 | 50.0 | 33.3 | 25.0 | 11.1 |
|  | Southeast | 12.5 | 25.0 | 11.5 | 5.9 | 37.5 | 19.2 | 18.2 | 7.4 |
|  | South | - | - | 33.3 | 16.7 | 100.0 | 37.8 | 33.3 | 13.7 |
| Congenital Toxoplasmosis | **Brazil** | 4.3 | 3.6 | 3.1 | 2.6 | 5.2 | 3.7 | 3.7 | 0.5 |
|  | Central-West | 2.2 | 2.6 | 2.3 | 0.8 | 4.6 | 2.4 | 2.3 | 0.8 |
|  | Northeast | 2.0 | 1.7 | 2.4 | 0.7 | 6.7 | 1.8 | 1.5 | 0.8 |
|  | North | 15.4 | 2.2 | 3.4 | 0.9 | 15.4 | 3.4 | 3.0 | 1.7 |
|  | Southeast | 3.9 | 4.5 | 3.2 | 2.7 | 6.3 | 4.3 | 4.4 | 0.9 |
|  | South | 12.5 | 4.8 | 6.1 | 4.8 | 30.0 | 10.9 | 8.1 | 5.6 |
| Total congenital infections | **Brazil** | 6.5 | 4.5 | 4.4 | 3.6 | 6.6 | 5.0 | 4.6 | 0.9 |
|  | Central-West | 8.3 | 2.5 | 2.8 | 1.3 | 8.6 | 3.8 | 2.9 | 1.9 |
|  | Northeast | 2.8 | 2.0 | 1.7 | 1.3 | 3.5 | 2.2 | 2.0 | 0.5 |
|  | North | 8.9 | 2.5 | 8.5 | 2.2 | 8.9 | 4.3 | 3.5 | 1.8 |
|  | Southeast | 6.4 | 5.0 | 4.5 | 3.5 | 7.2 | 4.8 | 4.5 | 0.7 |
|  | South | 23.9 | 12.4 | 10.0 | 9.3 | 38.2 | 18.4 | 16.3 | 7.2 |

***Source****: Developed by authors, with data extracted from SIH/SUS, considering the selected ICD-10 codes.*

** Number of hospitalizations with ICU use/total number of hospitalizations*

**S. Table 5.** In-hospital Mortality Rate

|  |  |  |  |  | **(2008-2024)** | | | | |
| --- | --- | --- | --- | --- | --- | --- | --- | --- | --- |
| **Infection** | **Region** | **2008** | **2016** | **2024** | **Min** | **Max** | **Mean** | **Median** | **SD** |
| Congenital Syphilis | **Brazil** | 0.4 | 0.2 | 0.1 | 0.1 | 0.4 | 0.2 | 0.2 | 0.1 |
|  | Central-West | - | - | 0.3 | 0.1 | 0.4 | 0.2 | 0.2 | 0.1 |
|  | Northeast | 0.8 | 0.1 | 0.1 | 0.1 | 0.8 | 0.3 | 0.2 | 0.1 |
|  | North | 0.4 | 0.4 | 0.2 | 0.1 | 0.6 | 0.3 | 0.3 | 0.1 |
|  | Southeast | - | 0.2 | 0.1 | 0.0 | 0.3 | 0.1 | 0.1 | 0.1 |
|  | South | - | 0.3 | 0.1 | 0.0 | 0.6 | 0.2 | 0.1 | 0.1 |
| Congenital Rubella | **Brazil** | 1.6 | 1.5 | 0.3 | 0.3 | 3.7 | 1.7 | 1.7 | 0.7 |
|  | Central-West | 3.9 | - | - | 3.9 | 11.1 | 6.2 | 5.1 | 2.2 |
|  | Northeast | 1.5 | 0.9 | - | 0.6 | 5.2 | 2.3 | 1.8 | 1.4 |
|  | North | 5.0 | - | - | 5.0 | 5.0 | 5.0 | 5.0 | 0.0 |
|  | Southeast | 1.4 | 2.9 | 0.4 | 0.4 | 4.7 | 2.3 | 2.6 | 1.1 |
|  | South | - | - | - | 9.1 | 20.0 | 14.6 | 14.6 | 5.5 |
| Congenital Cytomegalovirus | **Brazil** | 5.5 | 1.0 | - | 0.3 | 10.3 | 2.3 | 1.0 | 2.1 |
|  | Central-West | - | - | - | 16.7 | 25.0 | 20.9 | 20.9 | 4.2 |
|  | Northeast | 4.2 | - | - | 2.0 | 7.7 | 4.6 | 4.3 | 1.6 |
|  | North | - | - | - | 25.0 | 25.0 | 25.0 | 25.0 | 0.0 |
|  | Southeast | 8.3 | 2.2 | - | 1.3 | 11.1 | 4.5 | 2.2 | 3.5 |
|  | South | - | - | - | 12.5 | 12.5 | 12.5 | 12.5 | 0.0 |
| Congenital Herpes | **Brazil** | 8.1 | - | - | 1.9 | 12.9 | 4.8 | 3.4 | 2.9 |
|  | Central-West | - | - | - | 16.7 | 16.7 | 16.7 | 16.7 | 0.0 |
|  | Northeast | 16.7 | - | - | 3.4 | 21.1 | 10.7 | 11.1 | 5.3 |
|  | North | - | - | - | 0.0 | 0.0 | 0.0 | 0.0 | 0.0 |
|  | Southeast | - | - | - | 0.0 | 0.0 | 0.0 | 0.0 | 0.0 |
|  | South | - | - | - | 0.0 | 0.0 | 0.0 | 0.0 | 0.0 |
| Congenital Toxoplasmosis | **Brazil** | 0.4 | 0.2 | 0.1 | 0.1 | 0.5 | 0.3 | 0.3 | 0.1 |
|  | Central-West | 2.2 | - | 0.5 | 0.5 | 2.2 | 1.1 | 1.0 | 0.4 |
|  | Northeast | - | - | - | 0.1 | 1.7 | 0.8 | 0.8 | 0.6 |
|  | North | - | - | 1.1 | 0.3 | 2.0 | 1.2 | 1.1 | 0.4 |
|  | Southeast | - | 0.5 | 0.2 | 0.1 | 1.2 | 0.4 | 0.3 | 0.2 |
|  | South | - | - | - | 0.5 | 5.0 | 1.5 | 0.8 | 1.2 |
| Total congenital infections | **Brazil** | 0.7 | 0.2 | 0.1 | 0.1 | 0.7 | 0.3 | 0.2 | 0.1 |
|  | Central-West | - | - | 0.3 | 0.2 | 0.6 | 0.4 | 0.4 | 0.1 |
|  | Northeast | 1.2 | 0.1 | - | 0.1 | 1.2 | 0.4 | 0.3 | 0.2 |
|  | North | 0.5 | - | 0.2 | 0.2 | 0.5 | 0.3 | 0.3 | 0.1 |
|  | Southeast | - | 0.3 | 0.1 | 0.1 | 0.6 | 0.2 | 0.2 | 0.1 |
|  | South | - | - | - | 0.1 | 0.3 | 0.2 | 0.2 | 0.1 |

***Source****: Developed by authors, with data extracted from SIH/SUS, considering the selected ICD-10 codes.*

** Number of in-hospital deaths per 100 hospitalizations*

**S. Table 6.** Percentage of Hospitalizations that took place outside the patients' municipality of residence

|  |  |  |  |  | **(2008-2024)** | | | | |
| --- | --- | --- | --- | --- | --- | --- | --- | --- | --- |
| **Infection** | **Region** | **2008** | **2016** | **2024** | **Min** | **Max** | **Mean** | **Median** | **SD** |
| Congenital Syphilis | **Brazil** | 23.4 | 26.1 | 27.9 | 21.3 | 28.9 | 25.8 | 26.2 | 2.0 |
|  | Central-West | 20.2 | 24.0 | 26.1 | 16.9 | 33.2 | 24.8 | 24.4 | 3.2 |
|  | Northeast | 34.2 | 38.9 | 37.8 | 30.3 | 40.6 | 37.0 | 37.8 | 2.5 |
|  | North | 18.6 | 22.7 | 26.0 | 16.9 | 28.6 | 21.6 | 21.1 | 2.4 |
|  | Southeast | 13.1 | 17.6 | 18.2 | 12.3 | 21.7 | 17.3 | 18.1 | 2.3 |
|  | South | 22.2 | 22.7 | 32.1 | 18.9 | 32.1 | 23.5 | 22.5 | 3.0 |
| Congenital Rubella | **Brazil** | 43.1 | 43.0 | 27.9 | 27.9 | 54.3 | 39.5 | 41.1 | 5.7 |
|  | Central-West | 23.5 | 37.5 | 72.7 | 19.1 | 100.0 | 41.9 | 35.3 | 17.5 |
|  | Northeast | 57.6 | 58.0 | 46.9 | 36.2 | 76.0 | 55.8 | 56.9 | 8.0 |
|  | North | 15.0 | 50.0 | 50.0 | 15.0 | 100.0 | 44.0 | 50.0 | 17.2 |
|  | Southeast | 21.6 | 18.8 | 22.0 | 13.3 | 42.2 | 21.8 | 20.8 | 3.8 |
|  | South | 14.3 | 40.0 | 25.0 | 5.9 | 50.0 | 27.3 | 33.3 | 11.3 |
| Congenital Cytomegalovirus | **Brazil** | 30.9 | 20.4 | 33.7 | 17.8 | 33.7 | 25.8 | 26.2 | 3.6 |
|  | Central-West | - | 60.0 | 35.9 | 11.1 | 66.7 | 32.0 | 25.0 | 13.9 |
|  | Northeast | 45.8 | 13.9 | 44.8 | 10.0 | 45.8 | 31.6 | 34.0 | 9.6 |
|  | North | 33.3 | 33.3 | 100.0 | 14.3 | 100.0 | 48.5 | 50.0 | 19.6 |
|  | Southeast | 16.7 | 15.2 | 24.1 | 15.2 | 33.3 | 19.9 | 19.6 | 3.6 |
|  | South | 33.3 | 50.0 | 41.7 | 12.5 | 66.7 | 40.9 | 40.0 | 12.6 |
| Congenital Herpes | **Brazil** | 37.8 | 17.5 | 24.4 | 9.1 | 37.8 | 19.9 | 19.2 | 4.4 |
|  | Central-West | 25.0 | - | 50.0 | 14.3 | 50.0 | 32.9 | 29.2 | 11.5 |
|  | Northeast | 38.9 | 16.7 | 22.9 | 5.6 | 50.0 | 21.0 | 18.7 | 8.9 |
|  | North | - | - | - | 50.0 | 75.0 | 56.3 | 50.0 | 9.4 |
|  | Southeast | 50.0 | 25.0 | 23.1 | 10.0 | 50.0 | 24.0 | 24.3 | 7.1 |
|  | South | 40.0 | - | 27.8 | 17.2 | 100.0 | 38.1 | 33.3 | 14.1 |
| Congenital Toxoplasmosis | **Brazil** | 18.2 | 30.7 | 43.5 | 18.2 | 45.4 | 30.9 | 30.7 | 7.0 |
|  | Central-West | 8.7 | 28.9 | 30.3 | 8.7 | 42.0 | 26.7 | 30.3 | 8.7 |
|  | Northeast | 36.0 | 28.8 | 54.0 | 28.6 | 57.8 | 40.4 | 37.0 | 9.0 |
|  | North | 53.8 | 29.3 | 34.1 | 14.4 | 53.8 | 27.6 | 24.3 | 7.6 |
|  | Southeast | 12.3 | 27.7 | 32.5 | 12.3 | 33.5 | 26.4 | 27.7 | 4.2 |
|  | South | 18.8 | 41.3 | 51.5 | 12.9 | 53.1 | 34.9 | 36.8 | 7.9 |
| Total congenital infections | **Brazil** | 26.7 | 26.6 | 29.5 | 22.1 | 30.0 | 26.8 | 26.9 | 1.7 |
|  | Central-West | 18.5 | 25.2 | 27.3 | 18.5 | 33.5 | 25.0 | 25.2 | 3.7 |
|  | Northeast | 40.3 | 38.5 | 39.9 | 32.9 | 41.5 | 38.5 | 38.8 | 1.9 |
|  | North | 19.3 | 23.2 | 26.4 | 16.2 | 29.4 | 21.9 | 21.3 | 2.6 |
|  | Southeast | 14.2 | 18.4 | 19.8 | 13.9 | 22.3 | 18.4 | 18.8 | 2.0 |
|  | South | 20.6 | 25.0 | 34.3 | 19.4 | 34.3 | 24.4 | 23.8 | 3.4 |

***Source****: Developed by authors, with data extracted from SIH/SUS, considering the selected ICD-10 codes.*

** Number of hospitalizations outside the patients’ municipality of residence/total number of hospitalizations*

**S. Material – Statistical Analysis**

**1. DIFFERENCES BETWEEN REGIONS IN 2024**

**1.1. Hospitalization Rate**

| **Region** | **Mean** | **Standard Deviation** |
| --- | --- | --- |
| Central-West | 20.50 | 33.13 |
| Northeast | 33.58 | 52.02 |
| North | 2.60 | 3.89 |
| Southeast | 70.32 | 71.06 |
| South | 27.78 | 42.95 |

**ANOVA:** F(4, 20) = 1.447, p = 0.1
 **Conclusion:** NO significant difference between regions (p > 0.05)

**1.2. Mean Hospitalization Cost**

| **Region** | **Mean (US$)** | **Standard Deviation (US$)** |
| --- | --- | --- |
| Central-West | 142.27 | 80.06 |
| Northeast | 134.41 | 40.30 |
| North | 165.76 | 52.14 |
| Southeast | 227.71 | 95.28 |
| South | 456.24 | 270.12 |

**ANOVA:** F(4, 20) = 4.426, p = 0.05
 **Conclusion:** NO significant difference between regions (p = 0.05)

**1.3. Mean Length of Stay**

| **Region** | **Mean (days)** | **Standard Deviation (days)** |
| --- | --- | --- |
| Central-West | 7.56 | 3.07 |
| Northeast | 6.84 | 1.44 |
| North | 6.78 | 2.58 |
| Southeast | 6.78 | 2.30 |
| South | 8.78 | 2.89 |

**ANOVA:** F(4, 20) = 0.590, p = 0.1
 **Conclusion:** No significant difference between regions (p > 0.05)

**2. TEMPORAL TRENDS (2008-2024)**

**2.1. Hospitalization rate, by congenital infection**

| **Infection** | **2008** | **2024** | **ρ (Spearman)** | **p-value** | **Trend** |
| --- | --- | --- | --- | --- | --- |
| A50 Congenital Syphilis | 105.10 | 726.35 | 0.647 | 0.0048 | Increase |
| P35.0 Congenital Rubella | 24.64 | 13.40 | -0.186 | 0.4800 | Decrease |
| P35.1 Congenital Cytomegalovirus | 1.80 | 15.90 | 0.946 | < 0.001 | Increase |
| P35.2 Congenital Herpes | 1.20 | 5.10 | 0.750 | < 0.001 | Increase |
| P37.1 Congenital Toxoplasmosis | 9.40 | 83.40 | 0.993 | < 0.001 | Increase |
| All infections | 142.10 | 844.10 | 0.980 | < 0.001 | Increase |

**2.2. Hospitalization Rate, by region**

| **Region** | **2008** | **2024** | **ρ (Spearman)** | **p-value** | **Trend** |
| --- | --- | --- | --- | --- | --- |
| Central-West | 12.10 | 20.50 | 0.370 | 0.1 | Increase |
| Northeast | 39.66 | 33.58 | 0.578 | 0.01 | Increase |
| North | 10.08 | 2.60 | 0.458 | 0.1 | Increase |
| Southeast | 74.60 | 70.32 | 0.613 | 0.01 | Increase |
| South | 24.36 | 27.78 | 0.748 | <0.001 | Increase |

**2.3. Mean Hospitalization Cost, by region**

| **Region** | **2008 (US$)** | **2024 (US$)** | **ρ (Spearman)** | **p-value** | **Trend** |
| --- | --- | --- | --- | --- | --- |
| Central-West | 330.74 | 142.43 | -0.885 | < 0.001 | Decrease |
| Northeast | 283.03 | 134.52 | -0.961 | < 0.001 | Decrease |
| North | 395.53 | 165.93 | -0.598 | 0.002 | Decrease |
| Southeast | 277.59 | 227.97 | -0.870 | < 0.001 | Decrease |
| South | 411.27 | 456.56 | -0.941 | < 0.001 | Decrease |

Conversion rate: 2008, 1US$ = 1,83 R$ / 2024, 1US$ = 5,39 R$

**2.4. Mean Length of Stay, by region**

| **Region** | **2008 (days)** | **2024 (days)** | **ρ (Spearman)** | **p-value** | **Trend** |
| --- | --- | --- | --- | --- | --- |
| Central-West | 6.78 | 7.56 | 0.537 | 0.05 | Increase |
| Northeast | 10.22 | 6.84 | -0.760 | <0.001 | Decrease |
| North | 8.98 | 6.78 | 0.539 | 0.05 | Increase |
| Southeast | 7.50 | 6.78 | -0.689 | <0.001 | Decrease |
| South | 7.28 | 8.78 | 0.054 | 0.1 | Increase |

**3. DIFFERENCES BETWEEN CONGENITAL INFECTIONS IN 2024**

**3.1. Hospitalization Rate**

| **Infection** | **Mean** | **Standard Deviation** |
| --- | --- | --- |
| A50 Congenital Syphilis | 8.40 | 5.84 |
| P35.0 Congenital Rubella | 17.94 | 30.71 |
| P35.1 Congenital Cytomegalovirus | 19.44 | 22.33 |
| P35.2 Congenital Herpes | 7.40 | 7.65 |
| P37.1 Congenital Toxoplasmosis | 101.60 | 65.83 |

**ANOVA:** F(4, 20) = 6.770, p = 0.05
 **Conclusion:** NO significant difference between infections (p = 0.05)

**3.2. Mean Hospitalization Cost**

| **Infection** | **Mean (US$)** | **Standard Deviation (US$)** |
| --- | --- | --- |
| A50 Congenital Syphilis | 172.38 | 55.58 |
| P35.0 Congenital Rubella | 342.97 | 243.42 |
| P35.1 Congenital Cytomegalovirus | 218.03 | 187.45 |
| P35.2 Congenital Herpes | 285.57 | 227.9 |
| P37.1 Congenital Toxoplasmosis | 108.56 | 14.24 |

**ANOVA:** F(4, 20) = 1.417, p = 0.1
 **Conclusion:** NO significant difference between infections (p > 0.05)

**3.3. Mean Length of Stay**

| **Infection** | **Mean (days)** | **Standard Deviation (days)** |
| --- | --- | --- |
| A50 Congenital Syphilis | 9.08 | 0.19 |
| P35.0 Congenital Rubella | 6.98 | 1.21 |
| P35.1 Congenital Cytomegalovirus | 6.22 | 2.78 |
| P35.2 Congenital Herpes | 9.80 | 1.96 |
| P37.1 Congenital Toxoplasmosis | 4.66 | 0.63 |

**ANOVA:** F(4, 20) = 8.194, p = 0.01
 **Conclusion:** **SIGNIFICANT difference between infections (p < 0.05)**

**4. IN-HOSPITAL MORTALITY BY REGION (2008-2024)**

**4.1. Differences Between Regions**

| **Region** | **N** | **Mean (%)** | **Standard Deviation (%)** | **Min (%)** | **Max (%)** |
| --- | --- | --- | --- | --- | --- |
| Central-West | 26 | 4.08 | 6.42 | 0.10 | 25.00 |
| Northeast | 46 | 2.83 | 4.48 | 0.10 | 21.10 |
| North | 26 | 2.60 | 6.67 | 0.10 | 25.00 |
| Southeast | 45 | 1.35 | 2.18 | 0.00 | 11.10 |
| South | 23 | 2.83 | 5.40 | 0.00 | 20.00 |

**ANOVA:** F(4, 161) = 1.353, p = 0.1
 **Conclusion:** NO significant difference between regions (p > 0.05)

**4.2. Temporal Evolution of Mortality**

| **Region** | **2008 (%)** | **2024 (%)** | **Absolute Change (p.p.)** | **Relative Change (%)** |
| --- | --- | --- | --- | --- |
| Central-West | 1.22 | 0.16 | -1.06 | -86.9 |
| Northeast | 4.64 | 0.02 | -4.62 | -99.6 |
| North | 1.08 | 0.26 | -0.82 | -75.9 |
| Southeast | 1.94 | 0.14 | -1.80 | -92.8 |
| South | 0.00 | 0.02 | +0.02 | N/A |

N/A: not applicable

Relative change could not be calculated for the South because the initial value in 2008 was zero, making percentage variation undefined.
